# Lightdrum—Portable Light Stage for Accurate BTF Measurement on Site

**DOI:** 10.3390/s17030423

**Published:** 2017-02-23

**Authors:** Vlastimil Havran, Jan Hošek, Šárka Němcová, Jiří Čáp, Jiří Bittner

**Affiliations:** 1 Faculty of Electrical Engineering, Czech Technical University, Karlovo nám. 13, 121 35 Praha 2, Czech Republic; bittner@fel.cvut.cz; 2 Faculty of Mechanical Engineering, Czech Technical University, Technická 2, 166 07 Praha 6, Czech Republic; jan.hosek@fs.cvut.cz (J.H.); sarka.nemcova@fs.cvut.cz (Š.N.); jiri.cap@fs.cvut.cz (J.Č.)

**Keywords:** surface reflectance, bidirectional texture function, surface reflectance measurement

## Abstract

We propose a miniaturised light stage for measuring the bidirectional reflectance distribution function (BRDF) and the bidirectional texture function (BTF) of surfaces on site in real world application scenarios. The main principle of our lightweight BTF acquisition gantry is a compact hemispherical skeleton with cameras along the meridian and with light emitting diode (LED) modules shining light onto a sample surface. The proposed device is portable and achieves a high speed of measurement while maintaining high degree of accuracy. While the positions of the LEDs are fixed on the hemisphere, the cameras allow us to cover the range of the zenith angle from 0${}^{\circ }$ to 75${}^{\circ }$ and by rotating the cameras along the axis of the hemisphere we can cover all possible camera directions. This allows us to take measurements with almost the same quality as existing stationary BTF gantries. Two degrees of freedom can be set arbitrarily for measurements and the other two degrees of freedom are fixed, which provides a tradeoff between accuracy of measurements and practical applicability. Assuming that a measured sample is locally flat and spatially accessible, we can set the correct perpendicular direction against the measured sample by means of an auto-collimator prior to measuring. Further, we have designed and used a marker sticker method to allow for the easy rectification and alignment of acquired images during data processing. We show the results of our approach by images rendered for 36 measured material samples.

## 1. Introduction

The measurement of surface reflectance has received significant attention in the past as the reproduction of real world appearance is indispensable for many scientific and industrial applications of computer graphics. This includes predictive rendering that allows to match the rendered images from the software to be indistinguishable from the real world. This is important, in particular for the movie industry and manufacturing industries that produce expensive products such as cars, where the final look of a product is of vital importance and should match the output from the software during its design phase. For a single point, the surface reflectance, more specifically *bidirectional reflectance distribution function*, (BRDF) was formalised by Nicodemus et al. [1]. Its general spatial extension to parameterisation over a surface is called *bidirectional texture function* [2] (BTF), which unlike *spatially varying BRDF* (SVBRDF) allows us to capture fine details and non-local effects such as subsurface scattering, self-shadowing and inter-reflection on the mesoscopic scale. Due to these phenomena BTF is a more general representation than BRDF/SVBRDF and unlike them BTF does not necessarily fulfil the energy conservation law and Helmholtz reciprocity. A simple description of BTF is that it is a texture image parameterized by viewing and illumination directions, assuming a constant intensity collimated light source is used for illuminating the measured surface sample.

**Figure 1:**
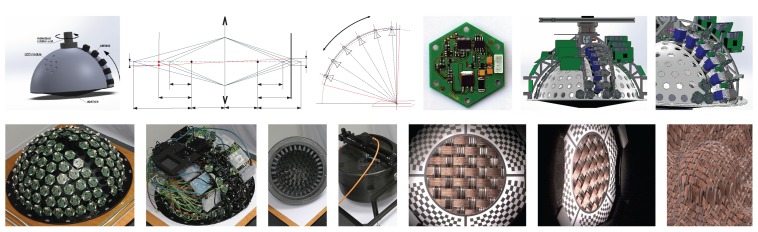
We propose a compact portable lightdrum for bidirectional texture function (BTF) and bidirectional reflectance distribution function (BRDF) measurements on site that uses the principle of a rotating light stage. It is suitable for real life application scenarios, including predictive rendering in cinema, design industry, etc. The project pipeline is shown, starting with our device concept to its design, construction, assembly, through measurement of a sample and finishing with a rendered 3D object covered by the measured material sample.

There are several challenges to obtaining a good technique to produce a realistic and accurate visual appearance of the rendered image using BTF that corresponds to the physical reality. We present such a technique starting from the conceptual idea through the design, construction, assembly and measurement to rendering images as outlined in Figure 1. The first challenge for any technique is the dimensionality of the measured and processed data. There are in total six dimensions needed for monochromatic BTF measurements (i.e., spatial position *x* and *y*, viewing direction $\omega_{o}$, and illumination direction $\omega_{i}$). The measured quantity $BTF(x,y,\omega_{i},\omega_{o})$ represents the visual appearance of a material sample for a surface point with coordinates (x,y) for the illumination direction over the whole measured sample $\omega_{i}$ and view direction $\omega_{o}$. The directions $\omega_{i}$ and $\omega_{o}$ are usually referred to in spherical coordinates, i.e., the incoming light direction $\omega_{i}$ is represented by a unit vector parameterized by $\theta_{i}$ and $\varphi_{i}$ using formulae $\omega_{i}=\left(\sin\left(\theta_{i}\right)\cos\left(\varphi_{i}\right),\sin\left(\theta_{i}\right)\sin\left(\varphi_{i}\right),\cos\left(\theta_{i}\right)\right)$ and $\omega_{o}$ by $\theta_{o}$ and $\varphi_{o}$.

**Figure 2:**
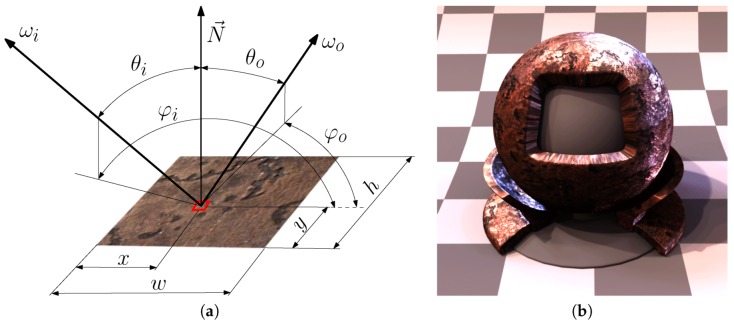
(**a**) A BTF parameterization corresponding to the ideal measurement arrangement of a planar sample of width *w* and height *h* of a texel at coordinates x,y. The illumination is provided by collimated incident light in the direction $\omega_{i}=\left(\theta_{i},\varphi_{i}\right)$ over the whole measured sample. $\vec{N}$ is the normal over the whole plane with the measured sample, $\omega_{o}=\left(\theta_{o},\varphi_{o}\right)$ is the outgoing light direction; (**b**) rendered image example, showing a 3D object covered with a measured BTF.

We illustrate the BTF parameterization that corresponds to an ideal measurement arrangement in Figure 2a and the example output with a rendered object in Figure 2b. Assuming collimated incident light to a sample, the BTF expressed as a ratio of incoming irradiance $E(\omega_{i})$ in $Wm^{-2}$ and outgoing radiance $L(x,y,\omega_{o})$ in $Wm^{-2}sr^{-1}$ is then unitless (i.e., $sr^{-1}$):

(1) $$BTF\left(x,y,\omega_{o},\omega_{i}\right)\left[sr^{-1}\right]=\frac{L(x,y,\omega_{o})}{E(\omega_{i})}$$

The high dimensionality of BTF data causes problems with acquisition and processing, as huge amounts of data must be measured and processed. The BTF is continuous but it can be measured only as a discrete function, the finer the discretization the better. Originally, BTF was measured only by stationary setups in laboratories using a gonioreflectometer principle, having a long measurement time (in the order of tens of hours).

In this paper, we propose a technically challenging concept for a BTF measuring device constructed as a portable light stage for use in real world on site scenarios. Our approach achieves high speed and accuracy. We extend the light stage concept by two means: the light stage rotates around its axis and the cameras move along the meridian. This way, we can position the camera in any direction to the measured sample from 0${}^{\circ }$ to 75${}^{\circ }$ in the zenith angle. The LEDs are mounted to the hemispherical skeleton and rotate together with the cameras, so their positions are fixed against the camera zero meridian. In summary, the contributions of our technique are:

- a novel design and construction of a working prototype of a portable BTF/BRDF measuring device that allows for its positioning against a sample by means of an auto-collimator, thus permitting on site measurement in real life scenarios with high speed and accuracy,
- the first portable device for on site measurements where a viewing direction (two degrees of freedom) to the measured sample can be set continuously and arbitrarily for up to maximum zenith angle 75${}^{\circ }$,
- a description of optical phenomena that limit either the spatial resolution or the size of the sample measured with BTF gantry,
- a marker sticker method for BTF/BRDF data acquisition which is used later in the data processing to align the data measured from the various camera directions,
- the documentation of our device construction by photographs of the individual parts and a step-by-step photographic documentation of the prototype assembly.

## 2. Related Work

Many applications of computer graphics require to achieve a predictive look of computed images for use in cinematography and industrial design, e.g., the car industry. The surface reflectance for more demanding applications can be formalised by means of BRDF for a single point. This four-dimensional function can be extended spatially by two other dimensions to BTF. This allows us to represent the effects arising from the light interaction of neighbouring elements on the surface, such as self-occlusion, masking, and scattering. While the BTF concept was originally proposed by Dana et al. [2,3] almost 20 years ago, there is still a big challenge to measure the data quickly and accurately.

As the problem of appearance measurement is difficult due to data dimension and size, there were proposed only a few approaches for measuring surface reflectance, in particular, for measuring BTF. In practice, only a couple of stationary and expensive devices for measuring BRDF for a spot on the surface are available on the market (as at May 2016). This hinders the practical use of the BTF concept in industries such as movie industry and virtual and augmented reality. There is a recent survey by Schwartz et al. [4] describing the options for measuring surface reflectance for computer graphics, including many details for the prototypes developed at the University of Bonn. Other older surveys are by Filip and Haindl [5], Weyrich et al. [6] and Mueller et al. [7], updated recently by Weinmann and Klein [8]. Most of the surface reflectance devices described in research papers, including those for measuring SVBRDF, utilise the Helmholtz reciprocity to halve the measurement time and sparsity of data. Such approaches are described in [9,10]. However, using Helmholtz reciprocity is inappropriate for BTF acquisition.

Portable instruments for both 4-dimensional BRDF and 6-dimensional BTF are rare due to the difficulty of putting the illumination and sensors into a small space, in particular, where the device is to be used for on-site measurement. The existing solutions are of very limited spatial and directional resolution. The first proposal for a portable instrument was outlined by Dana [11]. It uses an ellipsoidal mirror, a structured light source, and a beam splitter that allows for the separation of the incident light from the reflected light. Using a motorised XY stage which moves the gantry over the surface sample in scan-line order allows, in principle, the measurement of the surface reflectance variation over the whole surface. However, the set of input and output directions is limited by the shape of the ellipsoid and the beam splitter and to our knowledge this device has never been built.

Another principle uses a kaleidoscope and was proposed by Han and Perlin [12]. The idea is to use the reflections inside the kaleidoscope to achieve a variety of viewing and illumination directions. The separation of optical paths of incident and outgoing light is again achieved by a beam splitter. While the prototype with a 3-sided kaleidoscope was built in [12], the gantry allows for a limited and discrete choice of viewing and illumination directions and it achieved limited spatial resolution. The prototype was tested only under laboratory conditions.

Our approach is probably closer to the technique for measuring BRDFs in situ proposed by Ben-Ezra et al. [13] that uses a hemispherical setup, containing a set of LEDs. The measured sample was put in the centre, the LEDs were used for both the illumination and sensing of the light reflected from the sample. The stationary setup for measuring 3D objects, called a light stage, was proposed by Debevec at al. [14], where an object was put in the centre of a hemisphere containing light sources and cameras on its surface. This setup was used for measuring larger objects and seated humans for both the geometry and reflectance fields. This principle was adopted by Malzbender et al. [15] for polynomial texture maps providing low resolution reflectance acquisition. Mueller et al. [7] used this principle for the design of a stationary dome based setup for measuring BTF data. The details for the setup are in the recent survey [4]. Compared to the former goniometric based setups for BRDF and BTF including [2,16,17,18], this hemispherical setup with 151 cameras decreased the time for the measurement of BTF from 10 h to 90 min, achieving the acquisition rate 1600 Mpixels/s for different viewing and illumination directions. The setup was further revised by Schwartz et al. [19] by adding a rotary stage on which the sample is fixed and placing the cameras onto a meridian stage, which reduced the number of cameras used to twelve. However, while the setup can be disassembled into two parts and is portable in principle, its size and the necessity for calibration after reassembly makes its practical use difficult. Another interesting fully spherical stationary setup with cameras mounted on a rotating arc was proposed by Köhler et al. [20] and another possibly portable device with very limited directional resolution by Filip et al. [21]. More importantly, with the published approaches, it is not possible to measure the materials on site in any position and orientation. For this reason we do not provide a direct comparison with existing on-site approaches as in fact no such devices are available.

## 3. Lightdrum Overview

Our gantry design was motivated by several goals regarding its usage for real scenarios: (1) decreasing the time of measurement to the order of minutes; (2) allowing for true practical portability and on-site measurement, where no calibration is needed after the device is transported; (3) achieving high quality accurate data from the measurement that are useful for computer graphics and computer vision, and other goals such as (4) design robustness needed for measurement outside of laboratory conditions, unlike current BTF and BRDF measurement setups which were designed for laboratory conditions.

**Figure 3:**
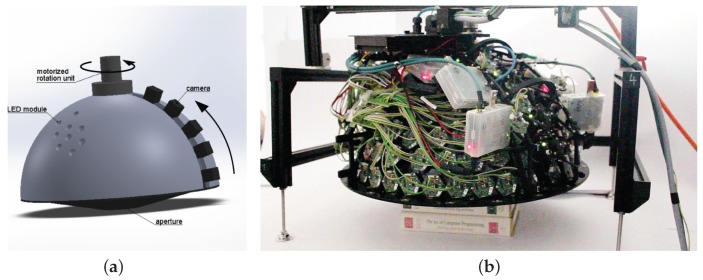
(**a**) The conceptual scheme of our device, consisting of one rotational motion around the z-axis of the hemisphere and one linear motion that approximates to the rotational movement of the six cameras along the meridian; (**b**) assembled device with electronics without carbon cover and servo motor during debugging.

To attain the desired goals, careful co-operative design involving several issues in software and hardware (mechanics, electronics and optics) had to be carried out. The conceptual scheme of our device is depicted in Figure 3a. We have achieved the goals by a new concept and by utilising recent results from the miniaturisation of camera and computer hardware. While we have used a light stage concept as the most natural solution for measuring surface reflectance, we have extended the light stage concept by adding two motions/degrees of freedom to allow for the arbitrary positioning of the camera over the measured sample. The device contains a hemispherical skeleton with custom LED modules mounted on it. The first degree of freedom is performed by a highly accurate rotational stage powered by a servo motor equipped with a gear box. This rotates the whole gantry over a measured sample. The second degree of freedom is achieved by a stepper motor over a linear rail, which moves six cameras along the meridian from the hemisphere pole ($\theta =0^{\circ }$) toward the equator (maximum $\theta_{max}=75^{\circ }$ in our case). This linear motion in a short range closely approximates the required circular motion.

The gantry is further equipped with an auto-collimator to allow for adjusting the gantry axis perpendicularly to the measured sample. The auto-collimator uses a laser and a small additional camera. The six cameras to acquire data are connected to microcomputers via fast universal serial bus (USB 3.0) that process the data from the cameras and allow for high dynamic range (HDR) image acquisition by multiple exposure with four images merged together. The six microcomputers are connected to another embedded microcomputer that controls the lighting and synchronises the LED modules flashes with the camera data acquisition. Further, this control embedded microcomputer (credit-card sized Raspberry Pi 2, information available at https://www.raspberrypi.org/) is connected to the camera for the auto-collimator, switches on/off the laser for the auto-collimator, and operates the stepper-motor and the servo motor.

All seven embedded microcomputers are connected via a miniature embedded network gigabit Ethernet switch. The remaining eighth port of the switch is used for data transfer from the device to external storage device for further processing. For safety reasons, the lightdrum uses low voltage power that is supplied from an external service power box which contains switching power supply units, a servo motor driver that operates the servo motor for rotational movement, and a network router to allow for data transfer to an external storage after the measurement. The lightdrum with the servo motor and the service power box are connected by three cables which allows for sufficient freedom of positioning the gantry during measurement.

**Figure 4:**
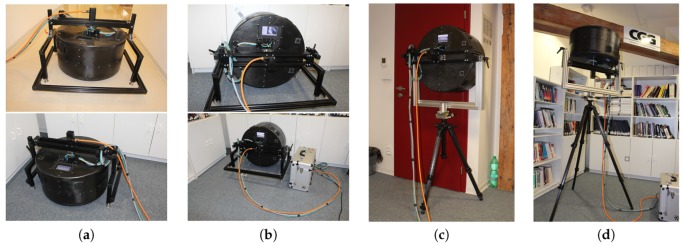
Instrument positioning: (**a**) on a desk/floor; (**b**) for measurement of a vertical wall near the floor; (**c**) for measurement of a vertical wall using a tripod; (**d**) for measurement of a ceiling using a tripod.

The device can be used for measurement in the lab preferably on a desk where the sample is placed. However, our design was motivated by the need for measurement outside the laboratory. We can categorise these outside laboratory situations into four different measurement positions for which we propose a solution as shown in Figure 4.

The assembled instrument, as depicted in Figure 4, can be seen from outside as a cylinder of diameter 600 mm and length 330 mm, which is prolonged to 520 mm when we include the geared servo motor. As such, the gantry allows for an arbitrary setting of the directions of the cameras over the sample so that the whole hemisphere up to zenith angle 75${}^{\circ }$ is covered, while the LED positions are fixed relative to the meridian line with the cameras. This achieves a balance between the fully settable four-dimensional positions for viewing and illumination, which is achievable by slow goniometric designs, and a completely fixed setup for both cameras and light source [7] that possibly achieves a very high acquisition speed, but is not portable and is costly and heavy as it contains many cameras.

## 4. Mechanical Design

The mechanical part of the device required substantial attention to both the design and manufacturing to achieve the required properties of the whole system. The device consists of two main parts: a light frame holder and a rotational drum.

The frame holder is made of standard high-tensile aluminium profiles and fasteners and allows for the positioning of the device over the sample located either in a vertical or horizontal position. We have built two versions: the first one for measuring a sample on the floor and the second one for measuring on walls and ceilings. The floor based frame holder (Figure 4a,b) consists of two U-shaped legs joined together by a horizontal bar which holds the lightdrum with the servo motor. The bar can be manually rotated so that an arbitrary tilt of the drum in one axis is set, both axes are then adjusted by changing the height of the legs. A servo motor with a harmonic gear box provides the rotary connection between the drum and the frame. The second frame holder (Figure 4c,d) for measuring on walls and ceilings is a simple U-shaped frame mounted on a heavy-duty tripod to maintain the stability of the system during the rotational movement. This also allows us to tilt the frame by rotating it around the tripod axis. By rotation in the U-shaped frame around the second axis we can set the gantry perpendicularly to the measured sample. Further, there is an XY stage mounted between the tripod and the U-shaped frame that allows us to finely adjust the mutual position of the gantry to the sample with the resolution of 0.02 mm.

The rotational drum contains the main body of the device as shown in Figure 4 and in Appendix D. It has a light-tight carbon mechanical and electromagnetic shielding for the plastic hemispherical skeleton with the LED modules, cameras and electronics. The rigidity is provided by an inner frame made of aluminium profiles. 3D printed holders for the LED modules are glued into circular holes in the hemispherical skeleton. The LED modules are attached to the holders by 3 screws with thick rubber washers. By tightening the screws, the direction of light is precisely adjusted. The cameras are equally spaced and fixed to a bar with a short linear ball guide-way at each end, oriented tangentially to the hemisphere. Near the pole, there is a stepper motor powered trapezoidal screw which slides the bar holding the cameras along two short linear guide-ways. This implements an approximately circular motion covering the whole span between the cameras. A prism beam splitter at the pole couples a laser module beam’s and an inspecting camera’s optical paths, forming an auto-collimator. For adjusting the laser module direction, the same mechanical solution as for the LED modules is used. Another conic carbon shield with a rectangular opening covers the drum from the bottom.

## 5. Cameras

Below we describe the camera selection and optical design. We also describe the mechanism for moving the cameras in the gantry along a meridian.

### 5.1. Camera Selection and Optical Design

The choice of camera and lens is driven by the spatial limitations of the gantry and the camera interfaces. In addition, the camera must be able to capture HDR images efficiently. In order to get a high data transfer rate from camera to disk we did a survey of small format cameras equipped with a USB 3.0 interface (up to 5 GBits/s) available in the first quarter of 2015. This included the following camera models: Point Grey FL3-U3-88S2C-C, Point Grey FL3-U3-32S2C-CS, Point Grey FL3-GE-28S4C-C, Basler acA2500-60uc, Basler ac2040-90uc, JAI GO-5000C-PMCL, and JAI 4200GE. Further, we considered various fixed focus lenses with a focal length from 8 mm to 75 mm.

For the optical design we considered all the combinations of these cameras and lenses. We computed which of them are capable of forming a sharp image with the necessary depth of field in an assumed range of minimum and maximum size of the gantry. In this respect our initial design goal was that we need to be able to transport the assembled instrument through 600 mm wide door.

**Figure 5:**
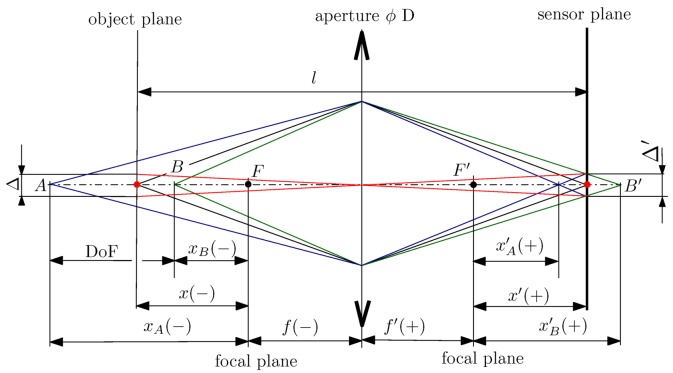
The geometry of thin lens imaging with a limited depth depth of field. Newtonian notation is used, i.e., object and image distances are measured from focal points. *x* ($x^{\prime }$)—object (image) distance; *A*—farthest point in focus; *B*—nearest point in focus; $\Delta^{\prime }$—pixel size; Δ—size of pixel projected on the object; *l*—object to image distance; DoF—depth of field; *f* ($f^{\prime }$)—focal length in object (image) space.

The input for optical design in such a case is the size of the gantry and the measured sample, making a tradeoff between the size of the measured sample (given the working distance from camera to the sample) and the achievable spatial resolution (a.k.a. pixel density), usually expressed in dots per inch (DPI). We want to get sharp images with a high spatial resolution. As the lowest camera in the gantry looks at the sample at a high zenith angle ($\theta_{max}=75^{\circ }$), the required depth of field (DoF) is nearly equal to the sample size *y* (exactly $y\sin\theta_{max}$). The optical design needs to fulfil the DoF required by the lowest camera for it to be plausible. The geometry of thin lens imaging considered in our design is shown in Figure 5.

DoF is the distance between the nearest (B) and furthest (A) points in focus. Images of these points are blurred spots with a diameter equal to the pixel size $\Delta^{\prime }$. DoF can be increased by closing the aperture stop of diameter *D*, but only to the point when the blur caused by diffraction at the aperture stop reaches the pixel size. In other words, the smaller the aperture stop the larger the DoF but also the bigger the diffraction spot. The equations that describe the relations between the spatial resolution, the camera properties and the sample size are interrelated. The formulae for deriving the DoF, based on the geometry of thin lens imaging and diffraction limit, are given in Appendix A.

**Figure 6:**
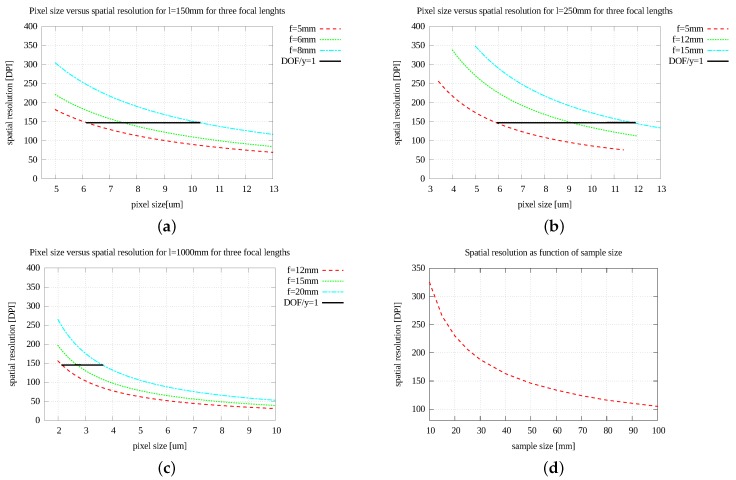
Spatial resolution in dots per inch (DPI) as a function of the sensor pixel size for the distance *l* of the object from the sensor (**a**) 150 mm; (**b**) 250 mm; (**c**) 1000 mm. Black line in all three charts indicates the condition DoF =y, for the sample size y= 50 mm; (**d**) Spatial resolution as a function of measured sample size computed for maximum zenith angle $\theta_{max}=75^{\circ }$.

Our design strategy is that we start with the distance *l* between the sample and the sensor given by the assumed size of the gantry (i.e., radius of the hemisphere), the sample size *y* and the objective focal length *f* and we search for the right F-number and pixel size Δ to achieve the highest spatial resolution calculated for the lowest camera. The spatial resolution is imposed by the required DoF because the camera views the sample at a high zenith angle. As can be shown by the formulae in Appendix A and in charts in Figure 6d, the maximum spatial resolution is determined by the sample size only, assuming DoF ≈ sample size *y*. No matter what the camera focal length or the sensor pixel size are, the maximum spatial resolution (in DPI) does not noticeably change (see Figure 6) and we can find the appropriate pixel size level for the target spatial resolution value. If we change the focal length *f*, only the pixel size changes and the DPI is kept the same. A change of the sample to camera distance *l* has almost no effect either (see Figure 6a–c). The relation between the sample size and the maximum spatial resolution is presented in Figure 6d.

For the design, we have used a fixed sample to camera distance l= 250 mm to obtain a relatively small footprint for the device which could still contain enough LED modules to be of practical value. We have considered all 6 small format cameras that allow for HDR acquisition listed above (pixel size on the sensor 1.55, 2.5, 3.69, 4.8, 5.5, 7.4 μm) and camera lenses with 16 mm C-mount (focal length 8, 10, 12.5, 16, 25, 35, 50, 75 mm), and the pixel binning 1×1, 2×2, 3×3, and 4×4. All the combinations were computed. We have opted for the size of measured sample of approximately 50 mm and the spatial resolution of 150 DPI as a practical trade-off between the spatial resolution and the sample size. This chosen design is achieved by the camera Point Grey FL3-U3-32S2C-CS (3.2 Mpixel, pixel size 2.5 μm with binning 4×4) and the lens Fujinon HF12.5HA-1B with focal length 12.5 mm.

We would like to emphasise that changing the distance of the sample to the camera *l* or increasing the camera chip size does not improve on the spatial resolution for a fixed sample size, as we have shown in the chart in Figure 6d. This is important for any future design of BTF measurement devices with fixed focus lenses, including the existing stationary gantries surveyed in [4]. To our knowledge, in the context of BTF measurement and instrument design, this relationship has never been highlighted and studied, although the depth-of-field is a severe limitation because the measured samples are viewed at high zenith angles.

### 5.2. Camera Positioning

The azimuthal movement of the cameras is performed by a geared servo motor rotating the whole drum over one of the two frame holders. To avoid inaccuracies, an appropriately sized harmonic drive gear box with practically zero backlash and high precision positioning is used. The servo motor uses a 20-bit multi-turn absolute encoder, so the positioning repeatability is very high. We calculated the error of this positioning to be 20 times smaller than the projected pixel size. This accuracy is more than sufficient because for each position of the servo motor we take all the images for all the LED modules from all six cameras before we rotate the lightdrum to the next position.

**Figure 7:**
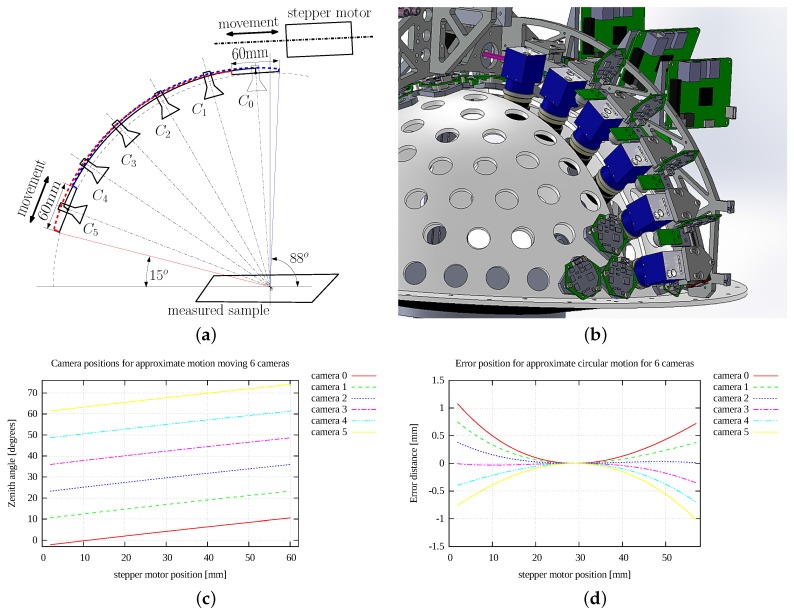
(**a**) The principle of six camera motion using two short linear guides 60 mm long carrying an arc with the cameras; the black circular arc is for the null position in the centre of linear guides, the blue dashed arc and the red dashed arc are the low and high dead centre positions of the motion, respectively; (**b**) solid drawing of the camera motion mechanism; (**c**) camera positions in relation to stepper motor position; (**d**) radial camera position error taken as the distance of camera axis from the centre of hemisphere as a function of the stepper motor position.

As the selection of circular guides is very limited, and additionally these are heavy, we have proposed a light-weight linear motion mechanism approximating the circular motion. The idea behind our solution is based on the assumption that we need the circular motion only in a limited angular range. As we use 6 cameras mounted on the arc set into required position by motion mechanism, its required angular motion range is only 12.5${}^{\circ }$. The principle of the light-weight motion mechanism used is shown in Figure 7a, its solid drawing in Figure 7b. It was described by Hošek et al. [22]. The idea of putting the cameras along the arc above a measured object was proposed by Tong et al. [23].

The proposed solution consists of two short linear guides mounted on the inner frame at the angle $\theta =4^{\circ }$ (first camera, close to the pole) and $\theta =67.5^{\circ }$ (last camera, closest to equator). The circular camera holder connects two carriages on both linear guides. We have optimised the angles and positions of the linear guides so as to minimise the deviation of camera axis with respect to the hemisphere centre, where the sample is located. The maximum deviation of the camera axis from the centre of the hemisphere is then only 1.3 mm for the selected sample size of 50 mm. The position for cameras is shown is shown in Figure 7c and the error from the proposed motion system against the ideal circular motion of cameras at the sample centre is shown in Figure 7d. This small positioning error is resolved by image processing as described in Section 11.

The top linear guide is driven by a stepper motor with a trapezoidal screw. The range of motion is 60 mm. The positioning error of the stepper motor is less than ±0.05 mm and there are more accurate linear motion mechanisms available if needed. The camera motion is designed so that each camera overlaps with its adjacent camera. Thus, a camera positioned in a one uttermost position can check the image obtained by its neighbouring camera in the opposite uttermost position of the stepper motor movement range. The number of cameras mounted on the circular arc were optimised along with the optical system, which is described in Section 5.1.

## 6. Illumination Units

Below we describe the design of an LED module to provide appropriate high quality illumination for reflectance measurement. We start with the selection of the most suitable LED model based on its spectral characteristics and lighting power. Additionally we describe the design of the whole LED module including its optics.

### 6.1. LED Selection

**Figure 8:**
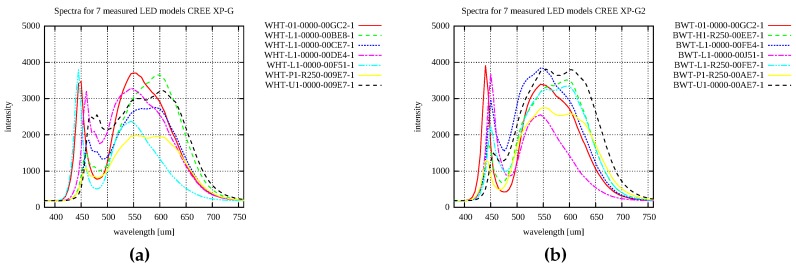
The spectral characteristics of evaluated light emitting diode (LEDs), **(a)** CREE X-lamp XP-G and **(b)** its newer version XP-G2, all performance groups into which producer classifies the LED one by one.

We need appropriate illumination in order to achieve a high fidelity of the colours in the measured data with the RGB trichromatic colour model used by the cameras in our proposed design. After general research of available products on the market and estimating the required illumination level we decided to use a high intensity white LED. First we studied the properties of various available LED models. After this initial study we selected 2 different high intensity white LEDs (CREE XP-G and CREE XP-G2) for further investigation. These LED models are classified to performance groups exhibiting different spectral characteristics and efficiency. As the information about their relative spectral power distribution was not available, we measured 14 different LEDs (7 models from the CREE XP-G group and the 7 from the CREE XP-G2 group). We measured spectra for 3 LEDs of each LED model to check for consistency of their spectra (in total 42 measurements). Within each of the two groups, there is a significant difference in spectra between the seven models, as shown in Figure 8. Fortunately, for any single LED model in either performance group, the variance of the relative spectral power distribution is insignificant.

**Figure 9:**
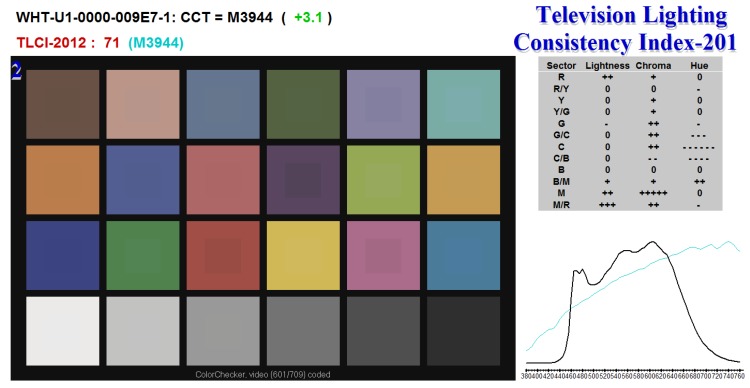
The Television Lighting Consistency Index (TLCI) performance of the chosen LED for 24 colour patches as the result of the TLCI application. The middle of each patch has a square of colour as evaluated for the tested LED while the remainder shows the reference colour.

As the colour rendering index [24] to characterise the quality of lighting is suitable for incandescent, fluorescent and high intensity discharge (HID) luminaires and it is generally not applicable for white LEDs [25], we adopted the newly proposed Television Lighting Consistency Index (TLCI) designed specifically for evaluating LEDs [26] for lighting in the context of television broadcast. It uses the whole simulated pipeline from a standardised television camera acquiring the image of a set of colour patches with known reflectance spectra illuminated by the luminaire being tested and a reference luminaire, processing the images including gamma correction and displaying the results on the output computer/video display. The quality of LED is then evaluated based on the difference in colours on the output for 24 colour patches. We have implemented the proposed approach and compared it with the reference TLCI implementation (available at the webpage https://tech.ebu.ch/tlci-2012). The evaluation of the selected LED model as the output from the publicly available software provided by EBU technical committee are shown in Figure 9.

We have further modified the TLCI methodology and have used the true spectral characteristics of the selected camera chip (Point Grey FL3-U3-32S2C-CS) instead of those in the standardised television camera model. In addition to 24 colour patches with known spectral properties we also used another set of 306 colour patches with known spectral characteristics and re-ranked all the measured LEDs. From the 14 LED models tested, the best LED model was a different to the one that the standard TLCI model would recommend. The LED with the best colour reproduction was CREE XPGWHT-U1-0000-009E7 with 80.6 lm output for input current 1500 mA. The change in the ranking by our modified TLCI evaluation is because the camera spectral characteristics are different for TV cameras and the camera that we considered for our evaluation.

We further tested the impact of the change of the electric current on the emitted spectra. This effect is difficult to predict but can affect the accuracy of colour reproduction. We therefore decided to measure reference data of surface reflectance for different currents and found that the spectra differ negligibly. Additionally, we measured whether the spectrum changes in time due to the warming up of the LED junction. Measurements taken with a fast 100 Hz spectrometer showed that the spectra are stable in time.

### 6.2. LED Modules

With the required goals in mind we have designed custom electronics on a printed circuit board (PCB) that contains the LED and other circuitry. In order to position as many LED modules as possible on the hemispherical surface, we have opted for a hexagonal shape of PCB with 6 mounting holes that allow us change its orientation if needed after mounting. The LED module allows us to set the flash time between 0 to 25 s and to set the current in a range of 0 to 1500 mA for lighting. The LED is positioned in the middle of the PCB. The design of the LED module allows for its operation with a continuous current of 500 mA. For higher currents it protects the LED from damage: on the opposite side of the PCB there is a temperature sensor which switches off the LED if the temperature passes a threshold. The LED can operate at maximum current for no more than 8 s before it must be switched off to cool down. As it will not be switched on again until all the other LEDs have been activated, this gives sufficient time for the LED to cool down properly. As shown later in Section 10.1 for the description of the measurement procedure, the maximum time the LED is switched on is much smaller (350 ms) so this serves mostly as a safeguard against damage in all circumstances (e.g., bugs in the measurement software controlling the LED modules). We did consider using heat sinks or aluminium based PCBs but with our carefully designed electronics it is not necessary thus avoiding the problem of extra weight on the device.

**Figure 10:**
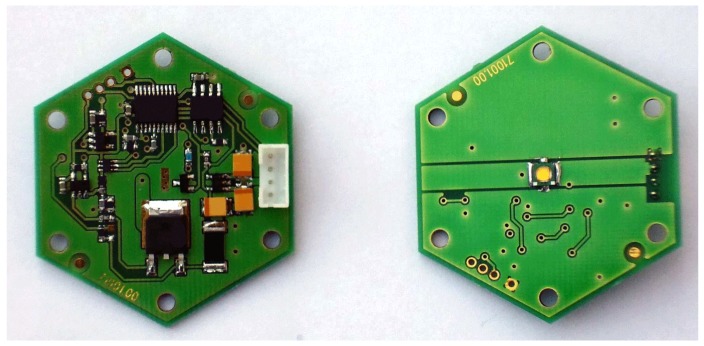
The prototype LED module on a hexagonal printed circuit board (PCB) with an outer diameter of 46 mm.

The LED modules are powered by 5 V and are equipped with a 4-line connector for power and communication. The set and execution commands are passed through a serial protocol over a RS485 communication line from the host microcomputer. Each LED module has a programmable ID. This way we can control one or many LEDs up to the limit of the power supply capability. The circuitry of the LED module consists of 24 parts of which 6 integrated chips are located on one side of the PCB. The second side of the PCB contains only an LED. The LED module also allows us to use an LED located outside the PCB. In this case the thermal protection cannot be used. The prototype of the PCB is shown in Figure 10.

**Figure 11:**
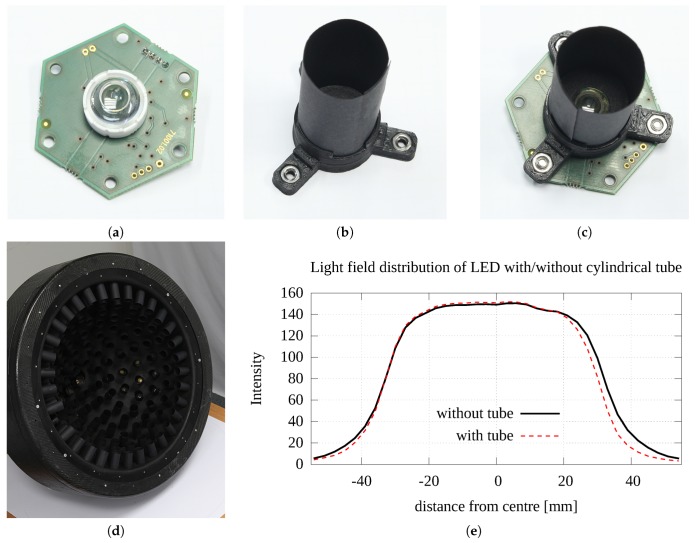
LED module: (**a**) PCB with lens; (**b**) tube mounted on the holder; (**c**) the assembled unit with three adjusting screws to get required direction; (**d**) photograph of the assembled PMMA dome showing the baffler created by the structure of tubes that effectively diminishes stray light inside the dome; (**e**) the illumination intensity distribution at 250 mm distance from the LED module with the tube and without it.

The PCB allows for mounting lenses up to a maximum diameter of 31 mm. We carried out thorough research on off-the-shelf lenses for the Cree XP-G LED and based on their directional emitting diagrams we preselected 5 lenses from different manufactures for evaluation. For these 5 lenses we measured their directional emission distribution, their illumination uniformity and the size of the area lit at the distance used in our device. As the most appropriate model we selected the lens LEDIL FA11905_TINA3-S made of PMMA (Polymethyl methacrylate), with diameter 16.1 mm, height 11.4 mm, and the angle at FWHM 15${}^{\circ }$. It achieves the most uniform illumination at the sample area at the distance used. The LED module with the lens mounted is shown in Figure 11a.

We equipped the module with an additional tube of length 40 mm and inner diameter 20 mm made of black paper as shown in Figure 11b,c. The length of the black paper tube was maximised so as not to vignette the light cone emitted from the LED module, so the uniformity of illumination at the sample area 60 × 60 mm${}^{2}$ is kept at 95%. As the assumed number of LED modules to be installed in the gantry was high, the use of the tubes minimises the unwanted scattering of light from the sample towards the hemisphere and back to the sample. In fact, the structure of black paper tubes inside the dome is a very effective baffler that decreases stray light inside the PMMA hemisphere as shown in Figure 11d.

The intensity of measured illumination at the sample distance is shown in Figure 11e. Also the lens redistributes the power from almost cosine directional distribution to only the required illumination area. As such, the LED module for maximum current 1500 mA reaches illumination level 12,500 lux at the sample distance.

### 6.3. LED Modules Distribution on Hemispherical Dome

**Figure 12:**
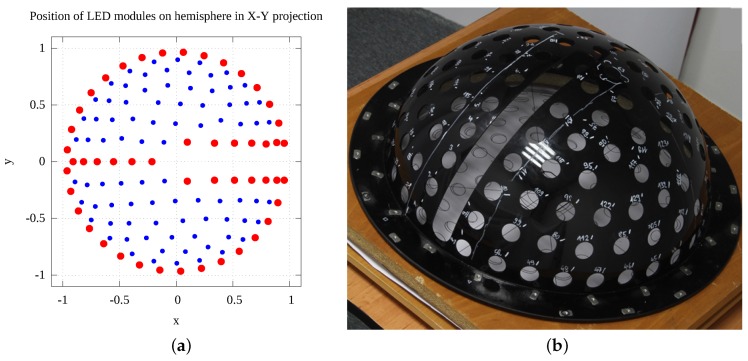
The distribution of 134 LED modules on the hemispherical skeleton. (**a**) the simulation model result shown projected onto an X-Y plane: bigger red points are for 50 deterministically set positions and 84 blue smaller points correspond to positions computed by randomised algorithm; (**b**) the manufactured skeleton from PMMA with holes for LED modules and slot for cameras. Note that 5 additional LEDs are mounted between the cameras and are not shown in this figure.

In our design the cameras are located along the meridian from the pole up to the required lowest zenithal direction ($\theta_{max}=75^{\circ }$) of an incomplete hemisphere. We had to distribute the LED modules described above on the hemispherical surface. We studied the properties of such a distribution with additional geometrical constraints, given by the LED module size and the zenithal slot for the cameras at the azimuthal angle $\phi =0^{\circ }$. While the problem can be understood as packing and/or sampling, it is not completely the case as our concern was also the uniformity of the modules’ distribution expressed by discrepancy of points on the sphere. A further consideration was that we wanted to accurately measure the specular reflection for highly reflective samples which requires the positioning of the cameras in the direction of ideal reflection from a luminaire. For all these reasons, we used a semi-deterministic algorithm to distribute the LED modules on the hemispherical skeleton. Some of the LED modules are positioned deterministically along the geometrical border of the slot with the cameras and around the border of the incomplete hemisphere (for zenith angle $\theta_{max}=75^{\circ }$). In addition, we added 6 LED modules positioned optically opposite the cameras based on the reflection at the mirrored sample, at an azimuthal angle $\phi =180^{\circ }$. The remaining LED modules are positioned on the hemispherical surface by a randomised algorithm using Lloyd’s relaxation [27] working with the minimum distance between two samples given by the LED module size. The randomised algorithm first puts the samples randomly where possible with respect to already positioned LED modules. Then, by further relaxation, it optimises the positions of the LED modules to make their distribution as uniform as possible. The sampling algorithm was run hundreds of times and the best solution with the maximum number of LEDs was used. The best solution has 134 LEDs tightly packed on the hemispherical skeleton with an outer radius of 234 mm. The distribution of the LED modules is shown in Figure 12. Further, an additional 5 LED modules were put between the cameras so that we can measure backscattering efficiently. These move together with the cameras along the meridian.

## 7. Other Measurement Issues

Above we have described the key components of the BTF measurement device. Below we describe the remaining parts required to make the instrument fully functional and operational on site.

The challenging problem for on site measurements is the positioning of the mobile gantry to the stationary sample to be measured. A stationary instrument can use a precisely machined sample holder provided with markers on the border that are used for registration of the acquired images, for example the one described in [4]. This method is not applicable to on site measurements. Even if the position of the cameras can be estimated from the measured data, as proposed by Vávra and Filip [28], the processing of acquired data can be prohibitively costly due to the repetitive run of Principle Component Analysis (PCA). Also the quality of image registration is then in principle difficult to guarantee. To achieve the same functionality for mobile devices measuring on site, we use two techniques. The first technique is the auto-collimator that allows us to set the gantry perpendicularly to the sample. The second is a marker sticker specially designed to allow for image registration by the information on its outer surface.

### 7.1. Auto-Collimator

**Figure 13:**
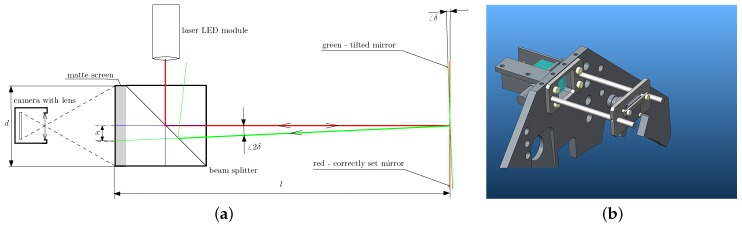
(**a**) Auto-collimator principle; (**b**) solid drawing of the auto-collimator assembly.

We have incorporated an auto-collimator used in optical devices to allow for the proper adjustment of the BTF measurement device against the measured sample. A mirror affixed tightly onto the sample must be used. If the gantry is put exactly perpendicularly to the mirror, the collimated light beam emitted by the auto-collimator is retroreflected to the same position. The principle is shown in Figure 13a and its construction variants were discussed in [29]. The auto-collimator design consists of a 25 mm right angle prism, a 25×25 mm${}^{2}$ cube beam splitter, a 25 mm square glass diffuser, a laser module as the source of collimated light and a miniature camera pointed at the glass diffuser. The three parts made of glass are cemented together. The beam from the laser module propagates through the beam splitter to the right angle prism and then goes to the mirror placed temporarily on the sample surface. The right angle prism is used only to minimise the height of the auto-collimator as the camera is then oriented horizontally. The use of an auto-collimator then increases the height of the gantry by only 25 mm. A solid drawing of the assembled auto-collimator unit with the camera is shown in Figure 13b.

For light travelling the distance 346 mm between the mirror and the ground glass, we can detect angle changes in the range $\pm 2.65^{\circ }$. The accuracy of determining the perpendicularity is relatively high. The image from a 5 Mpixel camera is shown on a display of size 600 × 480 pixels. It is theoretically possible to distinguish the angle deviation with an accuracy of up to 0.0027${}^{\circ }$. In our estimation, the realistically achievable perpendicularity of the gantry against the sample is then roughly 0.06${}^{\circ }$ as we are limited by the accuracy of calibration and the discernibility of the spots on the display. It is also restricted in practice as the mechanical resolution of the tilt adjustment is limited and we can never be sure the mirror is attached to the sample surface rigidly. Still, to our knowledge, it is the first use of an auto-collimator in the construction of a BTF measurement device, including stationary devices described so far in the literature.

The application of the auto-collimator is combined with the marker sticker method described in Section 7.2 and with the design of the whole device. To avoid outdoor light disturbance, the instrument is equipped at its bottom side with a conical cover with a rectangular measurement aperture of size 82×65 mm${}^{2}$ at the endpiece. The conical cover with its measurement aperture at the endpiece is placed 1 mm above the sample. This allows us to rotate the gantry over the sample without the influence of any outside light. The 1 mm gap between the endpiece and the measured sample also allows us to position the marker sticker and the thin mirror for the auto-collimator.

### 7.2. Marker Sticker Image Registration Method

To carry out registration for acquired images during BTF measurement we propose a marker sticker method similar to fiducial markers [30] used for augmented reality applications. We studied the literature and concluded that the design goals of the markers for augmented reality and for BTF measurement are different. The objective of fiducial markers in augmented reality is to find the markers reliably in the image and distinguish the information located in them with possible partial occlusion. In our case we know, with some uncertainty, where the marker is located: it is over the whole image, except for the measured sample in the centre of the image. Our objective is to get a design that maximises the measured sample area formed by a circle because the instrument rotates over the measured sample. At the same time we want to maximise the amount of information outside the circle to allow for image registration as there is an uncertainty in where the camera is positioned above the measured sample. To our knowledge, the design of a suitable flat marker has not been addressed so far as it is specific to the measurement of spatially varying surface reflectance on site. We propose an initial solution for this problem.

**Figure 14:**
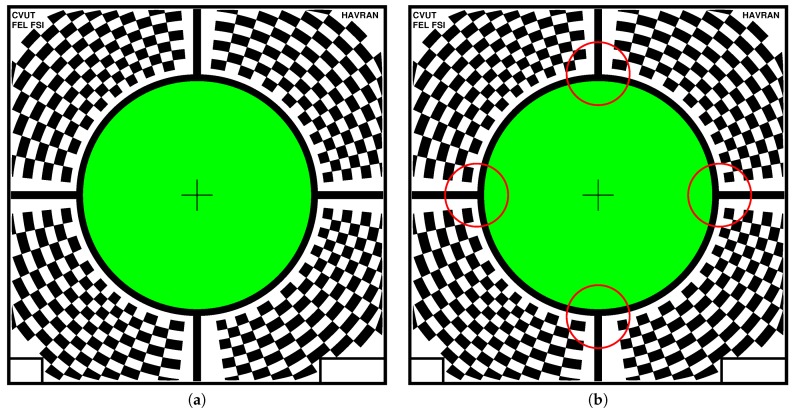
(**a**) Marker sticker design with a 51 mm diameter hole shown by the green colour; the size of marker sticker is 85 × 85 mm${}^{2}$, the angular distribution of chequerboard pattern is intentionally irregular; (**b**) Red circles mark the regions that allow for visual detection and checking of correctness of acquired images. This enables proper sample orientation in case of any image transformation by camera processing or for incorrect positioning the orientation of the gantry towards the measured sample.

Our marker sticker design is shown in Figure 14a and the source code generating the marker sticker pattern is in Appendix C. The marker sticker is a simplification of the sample holder used in stationary devices. It is made of a 0.10 mm thick aluminium foil of size 85 × 85 mm${}^{2}$ with a hole of diameter 51 mm in the centre where the sample being measured is located. The information for the registration is put as a black and white pattern on the non-measured border on the upper side of the marker sticker. The bottom side of the marker sticker has a thin coat of glue. The cameras on the spinning gantry aim at the centre of the sample. As the camera sensor chip is rectangular, there is space remaining outside the circular sample area in which we place the information needed for image registration. For this purpose we use a radial chequerboard pattern on the marker sticker outside the circular hole.

We designed a marker consisting of three functional parts. The first part is a black cross-hair used for centring the gantry over the sample. The second part is a 1.5 mm black and 1.5 mm white circular border around the hole to minimise the interference of light from the marker sticker surface to the sample. The black and white circular border can be used for determining the central position of the marker by algorithms finding a circle or ellipse within the image. The third part is a radial chequerboard pattern, algorithmically stretched in radial direction so that a unique pattern is formed in each quadrant of the marker sticker. This also allows us to detect unambiguously the rotational position of each image taken and also to detect the markers’ orientation by visual inspection as the chequerboard’s pattern size and distribution differ close to cross-hair, see Figure 14b. The marker sticker pattern is printed on the sticker foil with matte black and white paint by accurate silk-screen printing. The circular hole was made with a precision laser cutter. As such, the marker sticker is stable in shape and will not degrade over time assuming that the surface to which it is glued is locally flat and rigid. The 51 mm depth of field of the camera matches the dimensions of the marker. Due to the spinning of the gantry over the sample we can only measure a sample in a circular area and the information in the rest of the taken images is used for image registration. The size of the hole in the middle of the marker used for measurement is slightly smaller than the field of view of the camera. Therefore the pattern on the marker occupies the area that could have been used for sample measurement only a little. The rest of marker sticker area outside the circular hole for the sample is free for any use as the camera has a non-square sensor chip.

We describe the algorithm for image registration based on the marker sticker design in Section 11. The design of the irregularly spaced radial chequerboard pattern allows for a subpixel image registration of the measured data. We tested the functionality of the corner detection with OpenCV library, prior to the manufacturing of the marker sticker, to verify if the size of the features is sufficient for reliable detection under its assumed usage.

### 7.3. Measurement Setup Procedure

Before the measurement takes place the device has to be positioned over the sample to be perpendicular to its surface normal and to be at the position of the marker sticker. The most appropriate method we have found so far requires the manufacture of a thin but stiff plate mirror made of high quality steel, where a thin 0.1 mm glass mirror is either glued onto the 0.4 mm thin steel holder or the holder is a 0.5 mm thin metal plate coated by vacuum deposition. The suitable size of the mirror is therefore 40×500×0.5 mm${}^{3}$ to fit the design of the marker sticker described above. The whole procedure takes place before the measurement and consists of these six steps:

1. find a locally flat and appropriate position on the measured surface sample, verify it is accessible by the gantry,
2. glue on the marker sticker with a selected orientation that can relate to the structure of the measured surface sample,
3. temporarily fasten the mirror (by appropriate means such as power tape) so that the middle of the marker sticker contains the mirror, while the fastener of the mirror can be removed, and the border of the marker sticker with its centring marks is still visible. The 40 mm mirror width allows us to put the mirror at an angle of 45${}^{\circ }$ to the centring marks so that they remain visible.
4. position the measurement instrument, adjust both its perpendicularity and position against the sample using the mirror and centring marks on the marker sticker,
5. carefully remove the mirror fasteners, and slide the mirror away from the instrument’s measurement aperture. The 500 mm length of the mirror allows for easy manipulation of the mirror upon its removal, before the measurement takes place.
6. recheck the position of the instrument against the sample and start the measurement.

### 7.4. Electronics

**Figure 15:**
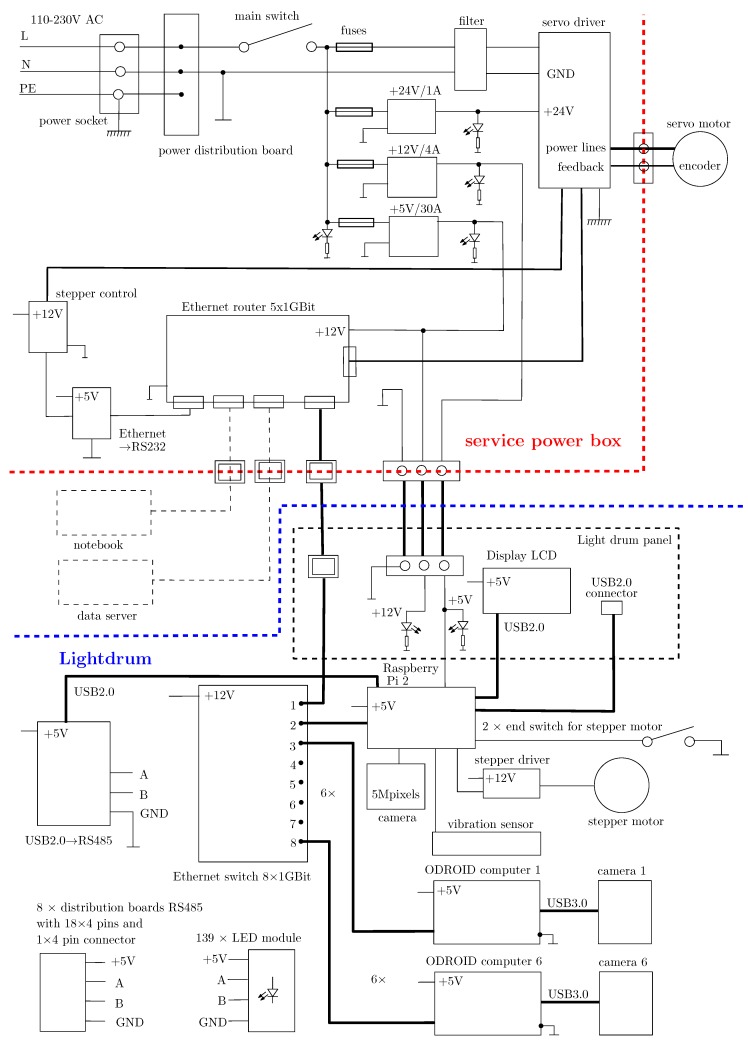
Electronics conceptual scheme for the instrument consisting of three main parts: service power box (separated by red dashed line), lightdrum itself (separated by blue dashed line), and servo motor. The three parts are connected by appropriate cables from service power box: power lines and feedback for servo motor, Ethernet cable and power cable (GND, +5 V, +12 V) to the lightdrum. Optionally, two other devices such as service notebook or data server to operate with the device can be connected via Ethernet.

The electronics conceptual scheme is shown in Figure 15. The lightdrum mounted on its frame is connected via three cables to the service power box that contains power supplies for 5, 12, and 24 V, the servo motor driver, and the network router to connect the device to the outer network. One cable transmits the power, the second one is the Ethernet cable for communication with the servo motor driver and outer network, and the third cable connects the servo motor to the servo driver. The separation into the lightdrum and service power box allows to decrease the weight of the gantry and to easier manipulate the lightdrum over the measured sample. The measurement is carried out in standalone mode (i.e., without using any personal computer). After the measurement it is necessary to transfer the acquired data to the external computer for further processing as all data are needed at one storage place.

The use of six USB 3.0 cameras requires us to use six embedded microcomputers that process and store the data acquired from the HDR cameras. We have chosen Hardkernel Odroid-XU3 board microcomputers (http://www.hardkernel.com) based on ARM quad core processors that are equipped with the USB 3.0 interface and active cooling. We use a Raspberry Pi 2 as the controlling computer of the whole instrument. This operates the whole measurement process: it masters the data acquisition carried out by the six Odroid-XU3 microcomputers with their cameras by triggering them each time an LED is switched on; it shows the information from the software application on the display; it controls the LED modules and it operates the servo motor and stepper motor to move the cameras over the sample. The Raspberry Pi 2 connects the small camera and laser that are used in the auto-collimator.

The Raspberry Pi 2 is also equipped with an external vibration sensor. During measurement it continuously checks if any disturbing motion has occurred and if necessary repeats the measurement. Further, it is necessary to connect all 139 LED modules by 4 wire cables using RS485. This is achieved by 8 distribution boards with a star topology, each board handling up to 19 LED modules. The connection of LED modules to the distribution boards was calculated so as to minimise the weight of cables in the system. Each cable length was adjusted prior to its mounting.

The seven embedded microcomputers communicate via a TCP/IP gigabit Ethernet network switch, where the last 8th port is used to communicate the data with the outside the instrument. This allows for the controlling of the servo motor drive and the transfer of data from the device to external storage.

## 8. Parts Production, Assembly and Debugging

**Figure 16:**
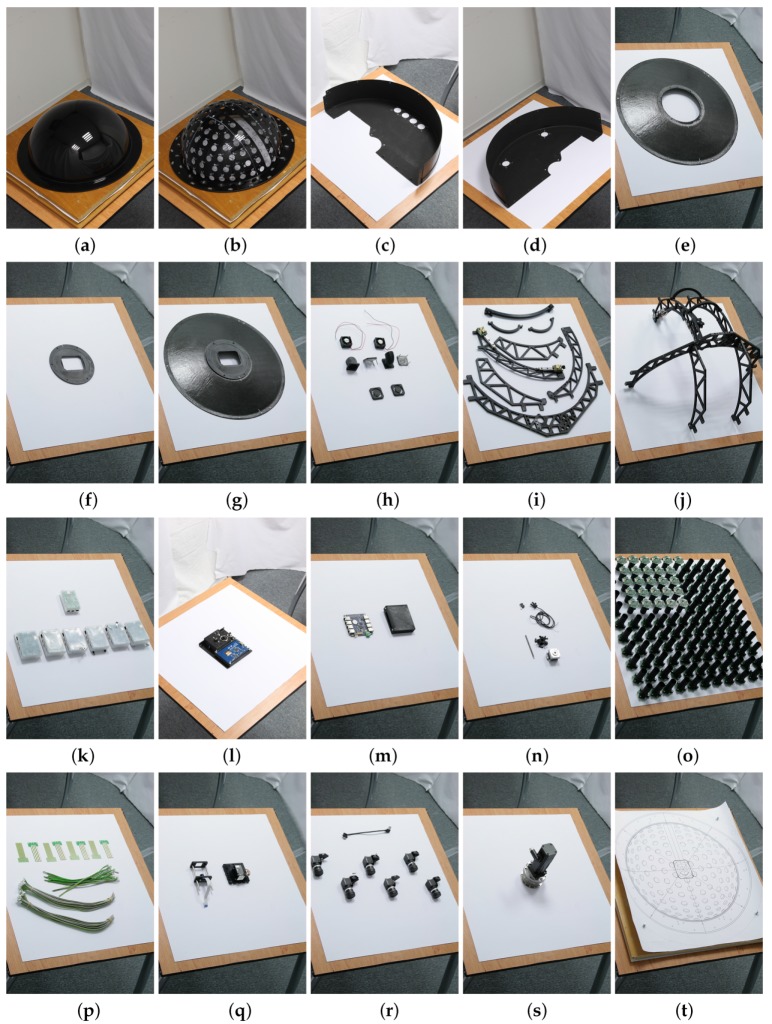
The individual parts of the lightdrum and the plan used for the initial adjustment procedure of the cameras and LED modules. (**a**) PMMA dome semi-product; (**b**) PMMA dome with CNC milled holes; (**c**) first top carbon cover part; (**d**) second top carbon cover part; (**e**) bottom carbon conic cover; (**f**) 3D printed endpiece with measurement aperture; (**g**) bottom carbon conic cover with mounted endpiece; (**h**) lightproof air ventilation parts; (**i**) inner aluminium frame and approximate circular positioning mechanism in pieces; (**j**) assembled inner aluminium frame and approximate circular positioning mechanism; (**k**) six Hardkernel Odroid-XU3 and one Raspberry Pi 2 microcomputers; (**l**) panel with display and connectors; (**m**) embedded gigabit Ethernet switch and its cover; (**n**) stepper motor with trapezoidal screw; (**o**) LED modules; (**p**) eight RS485 distribution boards and cables; (**q**) auto-collimator parts; (**r**) cameras with lenses and USB 3.0 cable; (**s**) geared servo motor; and (**t**) paper plan used for assembly and adjustment.

After carrying out the design and testing of some components, such as cameras and LED modules, all the components were manufactured. The hemispherical skeleton was made of the aircraft cockpit PMMA and the holes were made by a 5-axis CNC mill with a manufacturing accuracy better than 0.05 mm. The instrument consists of the following individual parts shown in Figure 16:

- the holding frame for measurement on the floor or the desk, or the tripod for measurement on the wall or ceiling, shown in Figure 4,
- the aluminium frame with geared servo motor (shown in Figure 16s) on which the lightdrum is mounted,
- the service power box with electronics (power supplies, servo motor drive, and gigabit Ethernet router), shown in Figure 4b,
- the outer carbon cover consisting of three parts, protecting the instrument from damage and disturbing external light, in Figure 16c–g,
- the inner aluminium frame construction that provides mechanical support and is mounted on the servo motor gear output, in Figure 16i,j,
- the approximate circular positioning mechanism with a stepper motor (Figure 16n) mounted on the inner aluminium frame, shown in Figure 16i,j,
- the PMMA dome mounted on the inner aluminium frame, in Figure 16a,b,
- the six cameras mounted on the approximate circular motion mechanism, in Figure 16r,
- the six embedded microcomputers for operating the cameras (Hardkernel Odroid-XU3), in Figure 16k,
- the Raspberry Pi 2 embedded controlling microcomputer (in Figure 16k), the panel with the display and connectors (in Figure 16l), the stepper motor driver and electronics for operating the laser module,
- the additional 5 Mpixel camera connected to the Raspberry Pi 2 and the laser module for the auto-collimator, in Figure 16q,
- the embedded gigabit Ethernet switch and its cover, in Figure 16m,
- the 139 LED modules (in Figure 16o) mounted on the PMMA dome,
- the cables inside the lightdrum; power, RS485 (in Figure 16p), and gigabit Ethernet),
- and the three cables between the external service power box and the lightdrum with the servo motor, shown in Figure 4.

**Figure A1:**
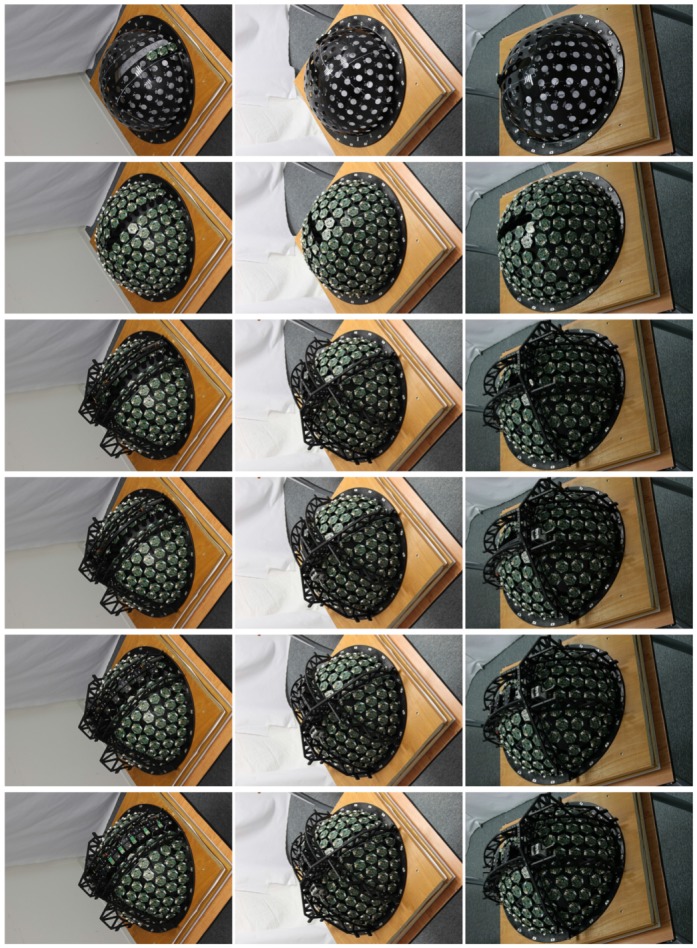
Assembly of lightdrum, step 1 to 6 from three different views. Row 1: populating first three LED modules; Row 2: populating all 134 LED modules; Row 3: mounting inner aluminium frame; Row 4: mounting stepper motor with trapezoidal screw; Row 5: mounting six cameras; Row 6: mounting five simplified LED modules inbetween cameras.

**Figure A2:**
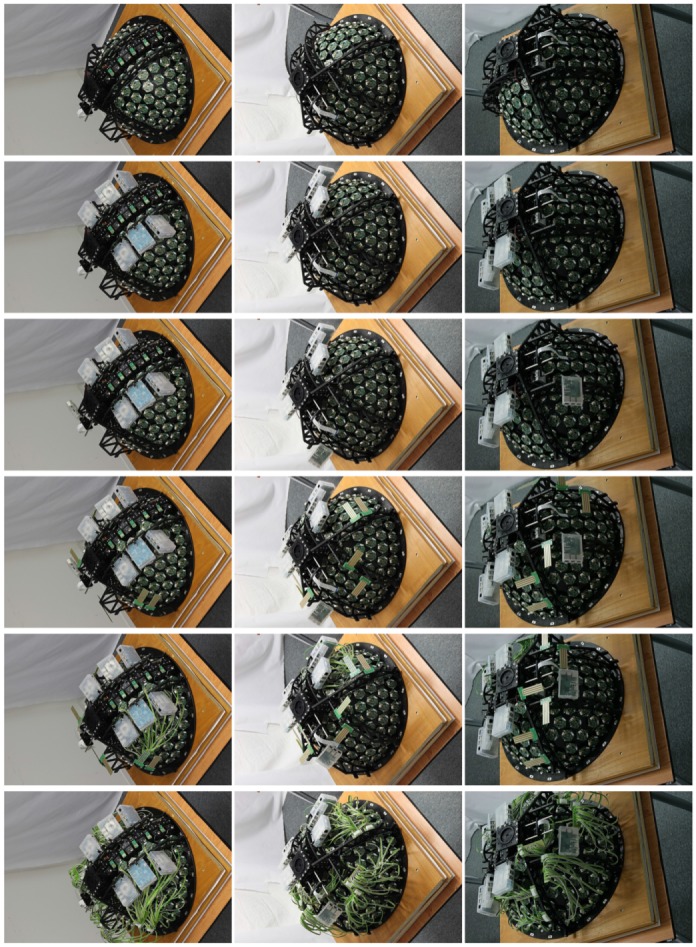
Assembly of lightdrum, step 7 to 12 from three different views. Row 1: mounting auto-collimator; Row 2: populating six microcomputers Hardkernel Odroid-XU3; Row 3: populating the microcomputer Raspberry Pi 2; Row 4: mounting the RS485 bus distribution boards; Row 5: connecting the first distribution board to LED modules; Row 6: connecting eight distribution boards to LED modules.

**Figure A3:**
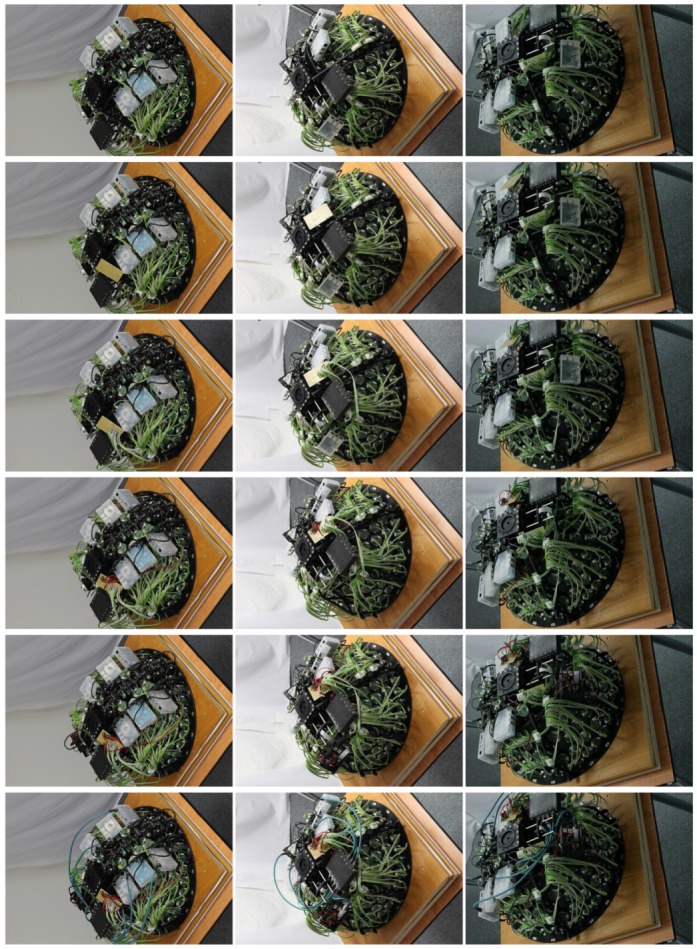
Assembly of lightdrum, step 13 to 18 from three different views. Row 1: mounting the gigabit Ethernet switch and connecting cameras with microcomputers by USB 3.0 cables; Row 2: adding the main RS485 distribution board; Row 3: connecting eight distribution boards to the main one; Row 4: connecting six microcomputers to the power; Row 5: changing the Raspberry Pi 2 cover, adding electronics and connecting stepper motor, laser module and camera for auto-collimator; Row 6: connecting six embedded microcomputers to gigabit Ethernet switch.

**Figure A4:**
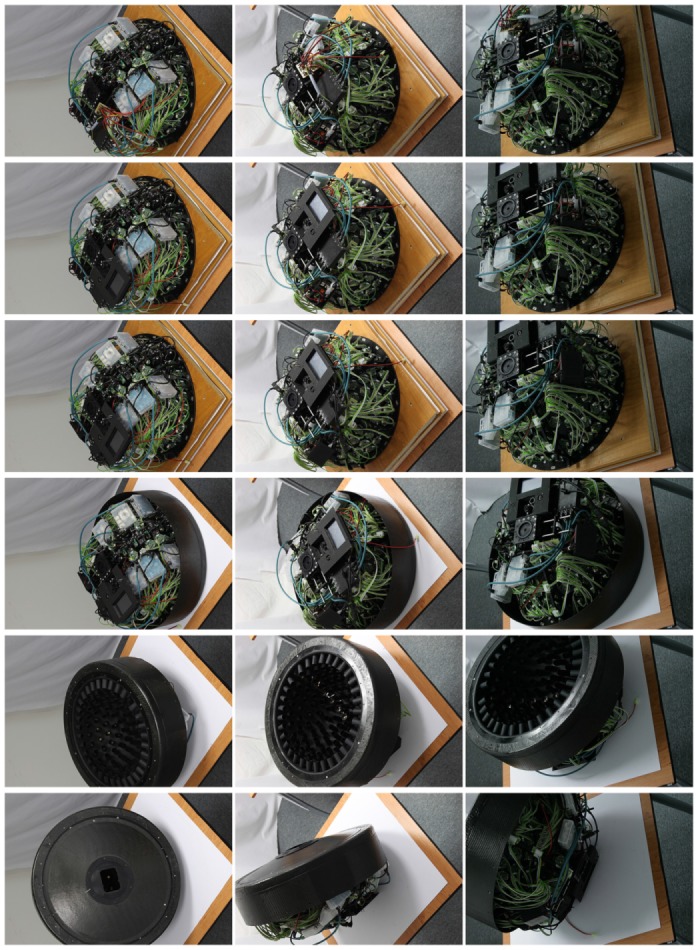
Assembly of lightdrum, step 19 to 24 from three different views. Row 1: adding the input power cable to the main socket; Row 2: connecting the input power cable to the power socket in the main panel with the display; Row 3: connecting the display to the Raspberry Pi 2; Row 4: mounting the carbon cylindrical cover part from bottom; Row 5: view to the gantry from bottom, the baffler is created by the set of LED module tubes; Row 6: mounting the bottom conic carbon cover, extended by 3D printed endpiece that contains the measurement aperture.

**Figure A5:**
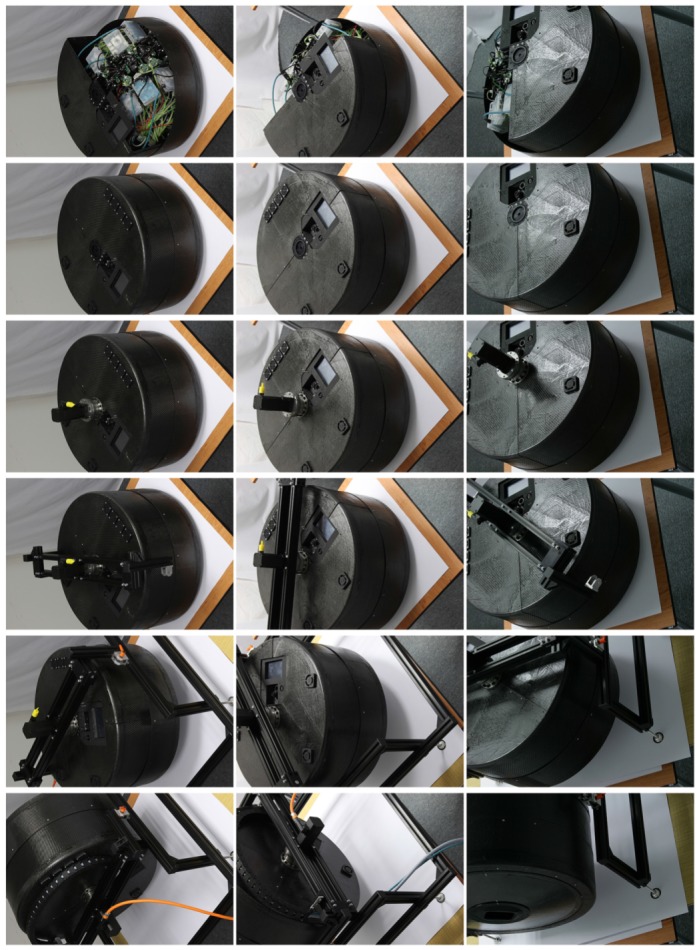
Assembly of lightdrum, step 25 to 30 from three different views. Row 1: mounting the left carbon top cover part; Row 2: mounting the right carbon top cover part and completing the lightdrum; Row 3: mounting the servo motor with gear box to the lightdrum; Row 4: mounting the aluminium profile holder onto the gear box; Row 5: mounting the lightdrum with the aluminium holder onto the holding frame for measurement of floor or on the desk, using two joints; Row 6: showing the same holding frame for measurement setup on the wall close above the floor.

The assembly is shown in a series of 30 images from three different views in Appendix D, in Figure A1, Figure A2, Figure A3, Figure A4 and Figure A5. The device requires careful assembly, paying attention to the cleanliness of the assembly process as metal splinters and particles remaining from the manufacturing could damage the electronics in the long term. Also the carbon cover protecting the device from outside illumination has to be protected by a lacquer as the carbon particles are highly conductive. Cleaning is best done by a vacuum cleaner and air pressure repeatedly applied during assembly.

The assembly of the device starts with (1) populating the LED modules to the PMMA dome and (2) adjustment of the LED modules’ direction towards the centre of the hemisphere; (3) The approximate circular positioning mechanism is then mounted on the inner aluminium frame part; (4) The inner aluminium frame is assembled and mounted on the PMMA dome; (5) The six cameras are populated on the arc of the positioning mechanism and (6) adjusted towards the hemisphere centre; (7) The distribution boards for the LED modules are mounted; (8) The 4-wire cable with RS485 bus is installed for each LED module; (9) The LED modules are then connected to a single wire guard connected to the power and embedded microcomputer controller for RS485; (10) The six embedded microcomputers are populated and connected to the cameras; (11) The gigabit Ethernet switch is mounted and connected to the embedded microcomputers; (12) The main panel with the display and two sockets (power line and gigabit Ethernet) is mounted and connected by cables to the power distribution board and to the gigabit Ethernet switch; (13) The stepper motor is mounted and aligned with the positioning system through the clutch and wired to the driver; (14) The camera and laser module for the auto-collimator are connected by cables; (15) The top carbon cover consisting of three parts is populated with a lightproof forced ventilator system and then mounted onto the gantry; (16) The final bottom carbon conic cover mounted is a cone extended by the 3D printed endpiece that contains the measurement aperture.

This carbon conic cover is provisionally mounted by three screws to the PMMA dome to allow adjustment as described below. The adjustment also requires another cover for the measurement aperture that has a centring mark indicating the middle of the aperture.

## 9. Adjustment

The device needs to be adjusted after assembly only once, the measurement then only needs us to position the instrument over the measured sample in the right way. The adjustment procedure has four steps.

In the first step we need to adjust the instrument’s aperture to be in the centre of rotation given by servo motor. The carbon conic cover with the rectangular measurement aperture can be moved in the XY direction on the rim of the hemispherical skeleton and fixed after the centre of rotation is found. The measurement aperture is centred by repeatably rotating the lightdrum by the servo motor and shifting the carbon conic cover until the centring mark on the endpiece is at the axis of rotation.

In the second step we adjust the LED modules so that they shine on the centre of the sample, using the three screws on each LED module holder. This may require us to dismount some parts to get access to some of the screws that mount the LED module to the mechanical part. The LED modules adjustment used a paper plan shown in Figure 16t to denote the centre of the aperture.

In the third step the cameras are first dismounted and then carefully focused to the right distance of the sample to be measured in the centre of the image. The A-stop is also carefully set and fixed. Then the cameras are remounted on the circular arc and positioned by the stepper motor to the null position given by the middle of the linear rails. Then each camera orientation is adjusted separately by three screws so that each one points at the centre of the sample, i.e., the centre of the hemisphere. This adjustment requires us to put the cover onto the measurement aperture so as to denote visibly its centre on the cover and hence on the aperture.

**Figure 17:**
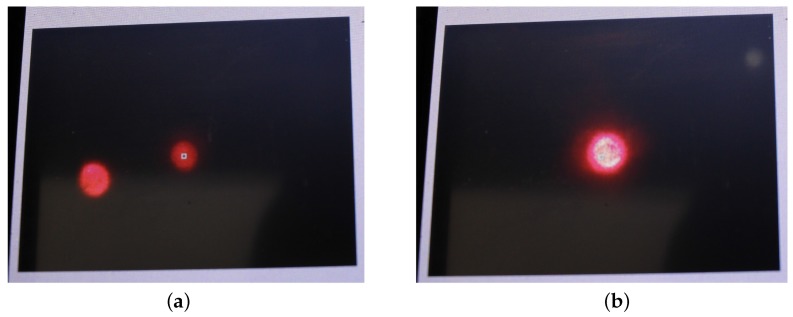
The photograph of the auto-collimator output at the display during operation: (**a**) the misaligned laser beams giving two spots; (**b**) aligned (i.e., collimated) laser beams giving one brighter spot.

In the fourth step the auto-collimator is adjusted. This is a slightly more difficult procedure, as we have to find the orientation of the lightdrum at the normal of a surface and adjust the laser module direction at the same time. At the beginning, we have neither the correct position of the device with respect to the surface normal nor the right adjustment of the laser module. The lightdrum is put onto the holding frame for measurement on the desk. The mirror is placed in the sample position on the desk. By repeatedly rotating the device to the positions 0${}^{\circ }$, 90${}^{\circ }$, 180${}^{\circ }$, and 270${}^{\circ }$, adjusting the device against the mirror in the boundary position and the laser orientation in the second direction, we get a converging procedure so finally the device is perpendicular to the mirror and the laser direction is properly adjusted too. At the rotation angle 0${}^{\circ }$ we tilt the device from the current point on the holding frame to the middle to direct the retroreflected beam from the mirror onto the matte screen observed by the camera. By adjusting the laser we move the beam spot to the centre. We then rotate the gantry to 180${}^{\circ }$ and repeat the procedure, first by tilting the gantry, second by tilting the laser. Then we rotate back to 0${}^{\circ }$ and repeat the procedure until we get a sufficient accuracy of adjustment. After adjustment with sufficient accuracy in this one direction we repeat the procedure for perpendicular directions 90${}^{\circ }$ and 270${}^{\circ }$. We then repeat both procedures in both directions $0^{\circ }$ ($180^{\circ }$) and $90^{\circ }$ ($270^{\circ }$) until we are satisfied with the convergence. The LED laser module direction at the auto-collimator is finely adjusted in two axes by the three M3 screws. The photography of the display with the image taken by the camera auto-collimator is shown in Figure 17 for two cases, misaligned and aligned beams.

## 10. Data Acquisition and Calibration

After the gantry is mechanically adjusted, we can acquire the data by measurement of a sample and carry out their radiometric and colourimetric calibration.

### 10.1. Data Acquisition

The data acquisition is initiated by the controlling software running on the Raspberry Pi 2. It sets the required position of the stepper motor and the null position of the servo motor. Then, for the initial position of the servo motor, the LED modules are switched on and off, one by one, and the HDR images are acquired on the Hardkernel Odroid-XU3 computers and saved to local storage disks on each board. The system is limited to roughly 3.5 HDR frames per second for one camera mainly because of the limited data throughput on Hardkernel Odroid-XU3 to internal/external storage. Each HDR image saved to the disk is composited from four camera images acquired for different exposures. The camera is running in HDR mode and does not need to set the parameters (i.e., exposure) for each taken image that minimises the acquisition time. Some time is needed for the synchronisation of the LED modules switching on and off and the cameras starting data acquisition. This is assured by a positive acknowledgement protocol that the LED module is switched on before the cameras start to capture the images. Similarly, the LED module is switched off after the last camera confirms the image capture.

**Figure 18:**
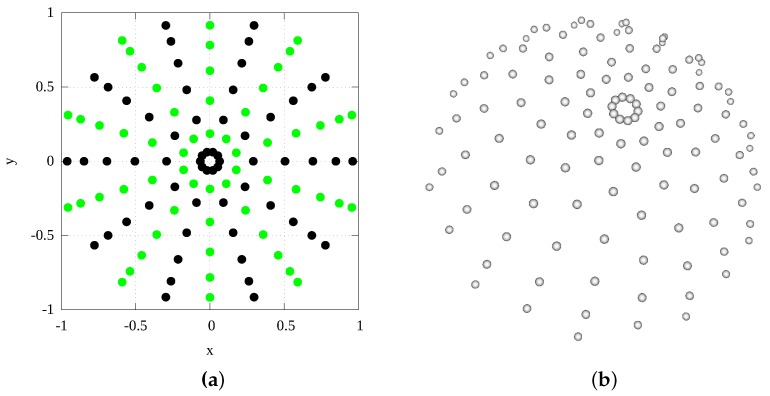
Visualization of 120 camera positions used for BTF data acquisition for 1020 s duration. (**a**) In XY projection from unit hemisphere The black dots represent camera positions for rotating servo motor clockwise, the green dots for rotating the geared servo motor anticlockwise; (**b**) 3D perspective visualization of camera positions as spheres.

While the number of camera directions can be set arbitrarily over the surface (with the limitation that all six cameras are always moved together) in practice we have to choose the number of images captured by the cameras as that influences the total measurement time. After initial testing of the system performance we decided to approximately match the number of camera directions on the hemisphere to the number of LED modules used in the gantry. The number of rotational positions given by the servo motor is 20, with the shift of the stepper motor for a half of those positions. The number of acquired images for this setting is 6×(10+10)=120. The lightdrum first rotates to 10 positions in steps of 36${}^{\circ }$ (0, 36, 72…, 324). It then moves the cameras using the stepper motor and rotates back using a different 10 rotational positions (342, …, 90, 54, 18). After it finishes the last data acquisitions, the servo motor rotates the drum back to the initial position ready for the next measurement. The camera directions on the hemisphere in projection to XY plane are shown in Figure 18.

The time used for positioning the stepper and servo motor is less than 5% of the total measurement time. The number of HDR images acquired for this measurement setting is 139×120= 16,680, the measurement time achieved for this setting is approximately 1020 s. The device allows us to use different illumination by changing the current to the LED modules to avoid over-saturation of HDR images, which is needed when measuring shiny materials. The saturated pixels are detected during acquisition and the measurement can be repeated with a smaller current to the LED module. We do not change camera exposure times as the camera is already running in HDR mode provided by camera firmware.

The number of images measured and hence the time and quality of measurement can be adjusted by taking either more or less HDR images. We decided to use this setting for the experiments, taking 16,680 HDR images during a single sample measurement, as this provides a tradeoff between the quality and the measurement time. The instrument performance depends on the camera speed, embedded computer power, storage speed etc. These will change in the future allowing higher acquisition rates in the same measurement time, or these will allow us to decrease the measurement time.

### 10.2. Radiometric and Colourimetric Calibration

We use two calibration targets for radiometric calibration: a sample of a diffuse black colour with a known reflectance (we used Acktar Ltd. Scatter Black${}^{\mathrm{TM}}$ material with 2% reflectivity) and a diffuse white calibration target Fluorilon-99W${}^{\mathrm{TM}}$ made by Avian Technologies (http://www.aviantechnologies.com). The white reference material made of sintered PTFE has an albedo of 99% for the visible spectrum and BRDF close to an ideal Lambertian surface (spectral and reflectance characteristics available at the company webpage http://www.aviantechnologies.com/products/coatings/fluorilon.php). Also four small patches, two of the black reference and two of the Lambertian white reference, of size 8×5 mm${}^{2}$ , were put at the corners of the measurement aperture to allow for the checking of the radiometric calibration for each taken HDR image. For the exposures of different LED currents the data for calibration targets have to be captured separately. To evaluate the surface reflectance from measurements the dataset measured for calibration targets corresponding to measurement have to be used. We use Equation (7) in Schwartz et al. [4] (pp. 7769–7771) and Equations (14) to (16) in [4] (pp. 7791–7793) that corresponds to Dana et al. [2] (p. 15). The radiometric calibration corresponds to the normalization of the data for each individual measured texel x,y for *I* of a measured sample, *W* of a reference white target, and *D* is the image measured without the sample, where the instrument aperture is opened in an unlit dark room. The following equation for radiometric normalization assumes linear response function of the camera:

(2) $$BTF\left(x,y,\omega_{o},\omega_{i}\right)\left[sr^{-1}\right]=\frac{L(x,y,\omega_{o})}{E(\omega_{i})}=\frac{(I\left(x,y,\omega_{o},\omega_{i}\right)-D\left(x,y,\omega_{o},\omega_{i}\right))a}{(W\left(x,y,\omega_{o},\omega_{i}\right)-D\left(x,y,\omega_{o},\omega_{i}\right))\pi },$$

where $L(x,y,\omega_{o})$ is the outgoing radiance in $Wm^{-2}sr^{-1}$, $E(\omega_{i})$ is the incoming irradiance in $Wm^{-2}$, $I(x,y,\omega_{o})$ is the intensity of the pixel of the image of a measured sample, $W(x,y,\omega_{o},\omega_{i})$ is the intensity of the pixel of a reference white target, $D(x,y,\omega_{o},\omega_{i})$ is the intensity of the pixel of an image with no sample present at the measurement aperture (unlit dark room), and *a* is the albedo of the reference white target (in our case a=0.99), assuming the same exposure time for taking all images.

The Equation (2) is formalised for monochromatic BTF, but our measurements are using trichromatic sRGB camera. Therefore it requires further colourimetric calibration of HDR images. The colourimetric calibration was carried out using the set of four colour calibration certified standards from Edmund Optics (red, green, blue, and yellow patch, the product number #56-079). The colourimetric calibration was carried out as described by Weinmann et al. [8] and Schwartz et al. [4].

## 11. Data Processing

After the measurement is complete, the acquired HDR images are transferred via network to the processing computer. This data transfer currently takes almost the same time as the measurement as we are limited by 1 GBit Ethernet and reading/writing storage speed to/from the disk. The raw measured data cannot be used directly in the application. First, they need to be corrected radiometrically and colourimetrically, as described above. Second, these still relatively raw data have to be processed to make them useful in applications such as rendering or computer vision. This data processing mostly covers the image registration and alignment.

We will describe such data processing of acquired images in detail. The acquired images have to be processed in the way used for BTF data as described in [4,31]. We modified the pipeline because we use the data from the marker sticker as described in Section 7.2 for image alignment.

The images need to be rectified as if they were viewed from the direction of surface normal (zenith angle $\theta =0^{\circ }$) and rotated as if they were seen from a single camera. These rectified images also have to be aligned with pixel or subpixel precision so that when we change from image to image, we get the images features aligned over the all pixels in the image.

Below we describe our image registration and alignment algorithm consisting of several steps. We document the functionality of these algorithmic steps using images for 5 different camera views out of 120 (20 rotations times 6 cameras), with the visible marker sticker pattern. For debugging and algorithm testing, we have used a simple grid 5 × 5 mm${}^{2}$, printed on standard white diffuse paper. The grid pattern is used in the images further on to demonstrate the algorithm steps. For the image registration algorithm we have assumed we know the basic orientation of the marker sticker to the gantry (0${}^{\circ }$, 90${}^{\circ }$, 180${}^{\circ }$, 270${}^{\circ }$). It is possible to visually check the gantry orientation on the acquired images, for the servo motor rotation angle 0${}^{\circ }$, using the four specific regions of the marker as shown in Figure 14b. In principle it could be fully automated, but we have not opted for this possibility in our current algorithm.

### 11.1. Step 1—Illumination Averaging

**Figure 19:**
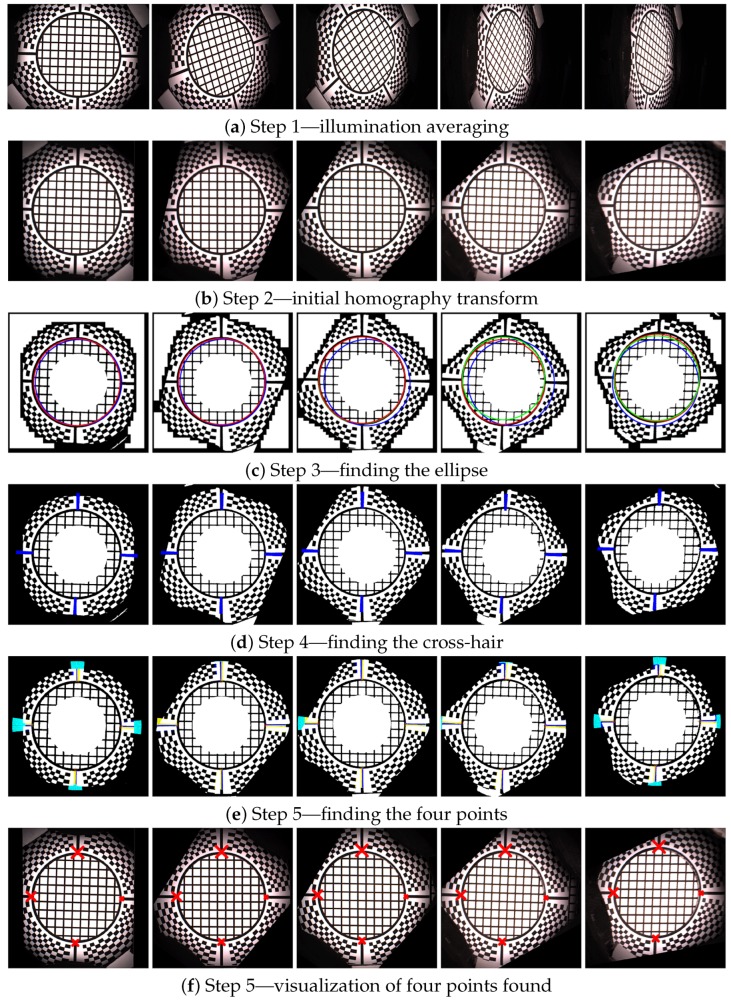
(**a**) Step 1—input data for camera #0 and rotation angle 0${}^{\circ }$, camera #2 and rotation angle 18${}^{\circ }$, camera #3 and rotation angle 36${}^{\circ }$, camera #4 and rotation angle 54${}^{\circ }$, and camera #5 and rotation angle 72${}^{\circ }$; (**b**) step 2—initial homography transform based on camera location; (**c**) step 3—finding the ellipse estimate fitting of the marker sticker circular border; (**d**) step 4—finding the cross-hair; (**e**) step 5 —finding the four points; (**f**) step 5—another more distinct view of the four points found.

We assume that all the images for one combination of the servo motor position and stepper motor position from a single camera have the same viewing geometry irrespective of the selected LED module used to illuminate a measured sample. This is only true if no vibrations occurred, which we check with the vibration sensor connected to the controlling computer. For each individual camera position we compute an average image from all the 139 images that were taken with a different LED module switched on. We then have, for the whole measurement of 16,680 images, only 120 images that need to be registered. The five example images computed with average illumination are shown in Figure 19a.

### 11.2. Step 2—Initial Homography Transform

Based on the camera location and direction in space in the ideal gantry given the servo motor and stepper motor position, with the sample positioned exactly in the centre of hemisphere, we compute the homography transform to a plane. The result of this transform, taking as input the image in Figure 19a is shown in Figure 19b.

### 11.3. Step 3—Ellipse Finding

In this step we use a randomised algorithm to first find a circle given by the black and white border of the marker sticker. To make this step more reliable the image is binarised to black and white. Further, the centre of the image is overwritten by a white in its circle. Similarly, the outer black regions that do not contain the chequerboard pattern are set to white. This procedure uses a simple algorithm that evaluates the image by rectangular regions and computes if most of pixels in these regions are black or white. The image regions containing at least 90% black pixels are set to white. Notice that the centre of the white circle is in the image centre while the circle given by the marker sticker circle is not centred due to the uncertainty of positioning of the gantry against the sample or an inaccuracy given by the gantry adjustment.

To find a circle on the marker sticker we generate a random circle with a centre in the range 15% from the image centre. For each iteration we also randomise the radius in a small range, as we have uncertainty of the gantry position to the sample, and the radius of the circle can vary as well. We take the circle that contains the most black pixels as the best match.

Further, we improve on the estimate by finding an ellipse instead of a circle. The initial ellipse centre and radii are those for the best matching circle found. The result of ellipse finding algorithm is visualized in Figure 19c. The initial circle that would correspond to a perfectly adjusted gantry and to an ideally positioned sample is shown in blue. The initially estimated circle is depicted in green. The final estimate of ellipse starting with the green circle is depicted in red. The images show that our algorithm does not compute perfect results in all cases, for the fourth and fifth columns. The perfect results are not needed as the inaccuracy of estimate are corrected in further steps.

### 11.4. Step 4—Cross-Hair Finding

Based on the estimate of the best fitting ellipse in the previous step, we rotate four conical black regions around the ellipse border. We find the best fitting of these conical black regions to the image, separately for each region. They correspond to the centring marks of the cross-hair. We again use the binarised image as the input. Each conical region is matched individually in a small range 15${}^{\circ }$ around its standard position (0${}^{\circ }$, 90${}^{\circ }$, 180${}^{\circ }$, 270${}^{\circ }$). The procedure outputs four deviation angles from the ideal setting. The visualization of this fitting is shown in Figure 19d, the found regions are in blue.

### 11.5. Step 5—Four Points Location

The next step is to detect the four points given as the intersection between the cross-hair and the black marker sticker circle around the hole. After some experiments we have come up with a robust version of an algorithm for this task. The algorithm starts with the position of the cross-hair found in the previous step, gradually decreasing the radius from large to smaller values. It verifies on the circular arc, given by the radius, that there is a sequence of white, black, white pixels. If not, it decreases the radius and starts again until it finds such a sequence. It may happen that the initial radius is too large and the evaluated circular arc does not intersect the cross-hair rectangle. Once such a radius of the arc is found and the cross-hair rectangle is located, the algorithm tracks it in the image towards the border of the ellipse found in step 3 by gradually decreasing the radius of the circular arc. Once the pattern on the circular arc changes from the sequence white-black-white to only black, it determines the previous pixel in the middle of the arc as the one to be the intersection of the black circle border and the cross-hair. The visualization of the algorithm search is shown in Figure 19e, the four points found are shown in Figure 19f. The four points of the reference marker sticker and the four points found are used to compute the improved homography transform.

### 11.6. Step 6—Chequerboard Fitting

**Figure 20:**
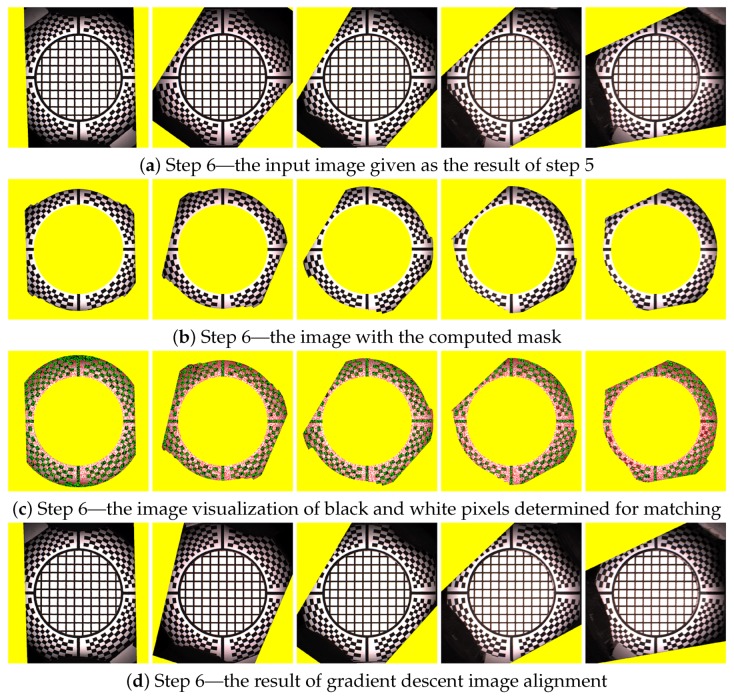
Step 6 of the algorithm. (**a**) Input image; (**b**) mask over the input image; (**c**) visualization of black/white classification of the masked input image by red and green marks; (**d**) the images from the homography computed by gradient descent search.

The four points found in step 5 are used to make a new homography transform that better aligns all 120 averaged images together. The result of this alignment is shown in Figure 20a and is used as the input in the step 6. By yellow colour we denote the region where we have no data from the camera when we apply the currently best known homography transform. We compute the mask for the part of the image where is no radial chequerboard pattern and also we cover the assumed position of the hole with the measured sample. The image with the mask applied, containing only the marker sticker pattern, is shown in Figure 20b. We compute the threshold from the histogram and use a subset of pixels to determine if they are black or white. The subset of 10% of all pixels evaluated is taken at precomputed random positions. Using only a subset of all pixels accelerates this algorithmic step. This is depicted in Figure 20c. The denoted white and black pixels are then fitted against the reference pattern in Figure 14a using a gradient descent search [32] applied to the homography matrix elements (8 degrees of freedom) to find the best match in the image. The result of the improved matching is shown in Figure 20d.

### 11.7. Step 7—Chequerboard Subpixel Fitting

**Figure 21:**
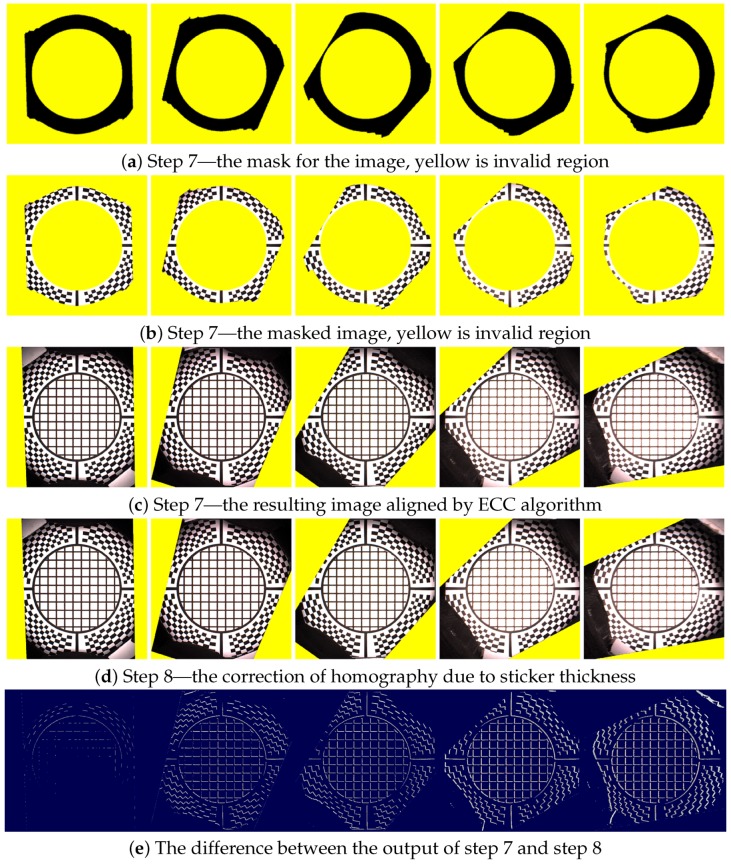
Completing image registration: (**a**–**c**) step 7—image alignment by ECC algorithm; (**d**) step 8—the image after the homography change due to the sticker thickness; (**e**) The difference image between images of step 7 and 8.

Although it could seem that the image registration from step 6 or even from step 5 is acceptable, the accuracy of image alignment is not sufficient yet as we aim for subpixel accuracy. By completing step 6 we approached close to the perfect homography transform but not close enough. To achieve the subpixel accuracy we perform parametric image alignment using the enhanced correlation coefficient maximisation algorithm (ECC) [33] implemented in OpenCV library [34]. First, we again create the image mask as in the step 6. The input image of the step 7 is the output of the step 6 and is shown in Figure 20d. The reference image is given by marker sticker pattern in Figure 14. The mask computed from the input image is shown in Figure 21a.

The mask applied to the input image is shown in Figure 21b. The input image, the mask, and the reference image are passed to the ECC algorithm that computes another correction homography with subpixel accuracy. The result of the ECC algorithm is then shown in Figure 21c.

### 11.8. Step 8—Marker Sticker Thickness Correction

In the step 7 we aligned all 120 images on the plane given by the marker sticker paint on its the top side, using the image features contained in the radial chequerboard pattern. This is however not the plane corresponding to the measured sample, as the aluminium foil including the glue is about t=0.15 mm thick. For the cameras viewing the sample at the direction close to the normal this is not important. But this shift on the measured sample increases with the camera zenith angle *θ* ($\theta =0^{\circ }$ for surface normal) with a factor of δ=ttanθ. We obtained the homography transform for the best fitting from step 7 on the top side of the marker sticker. In this step we correct the homography to compensate for the marker sticker thickness. We start by computing the correction homography transform by taking the 3D points corresponding to the intersection of the cross-hair with the black border (shown in Figure 19f but for the result of step 7), however, we shift these four points downwards towards the sample by the sticker thickness to the outer sample plane (i.e., bottom marker sticker plane). These four new points reprojected to the image plane and the four points without any shifting give the correction homography matrix. The image alignment is close to perfect at the top side of the sticker (Figure 21c) as the output of step 7, while the final image alignment is corrected so to be at the top of the sample in Figure 21d.

For the camera at the angle $\theta_{max}=75^{\circ }$ this effect becomes clearly visible and the shift corresponds to about δ=0.56 mm in the sample centre. For our chosen spatial resolution 150 DPI it corresponds to shift 3.3 pixels. The difference between the images before and after the sticker thickness correction is shown in Figure 21e.

### 11.9. Step 9—Data Transform and Processing

As the result of step 8 we obtain the homographies for all 120 camera directions over the sample that align the acquired images to BTF data. We then transform all the measured 16,680 images by the corresponding homographies. We apply the homography transform to the relevant part of the image to get a square image as the output. This corresponds in our case to a square region of about 35.5×35.5 mm${}^{2}$ on the measured sample. The output images have a pixel resolution of 200×200 pixels (in 150 DPI).

## 12. Results

We have manufactured, assembled and debugged the BTF measurement device as described in previous sections. The software for adjustment as described in Section 9 was written and all the adjustments were carried out. During the debugging phase of the gantry, to dissipate heat, we had to equip the lightdrum with an air cooling system with low noise ventilators with lightproof covers so that their function does not influence the measurements as some ventilators could vibrate to such a degree as to make the measurement impossible. The summary of properties of our BTF measurement instrument, together with the properties of the two dome based setups detailed in the survey paper [4], is given in Table 1.

### 12.1. Software

We have written the software for measurement with a GUI for the microcomputer Raspberry Pi 2 and also another piece of software for the Hardkernel Odroid-XU3 that communicates with the HDR camera. The software is written in C++ and runs under the Linux operating system. We have implemented and debugged the communication between the controller and the six microcomputers. The data are stored during measurement on the memory of Hardkernel Odroid-XU3 microcomputer. The acquired images from the six cameras are also possible to transfer to the Raspberry Pi 2 and further to the gantry display to allow for adjusting the gantry against the sample. Also the data from the additional camera of the auto-collimator can be displayed for setting up perpendicularity of the gantry against the sample, as shown in Figure 17.

### 12.2. Measurements

**Figure 22:**
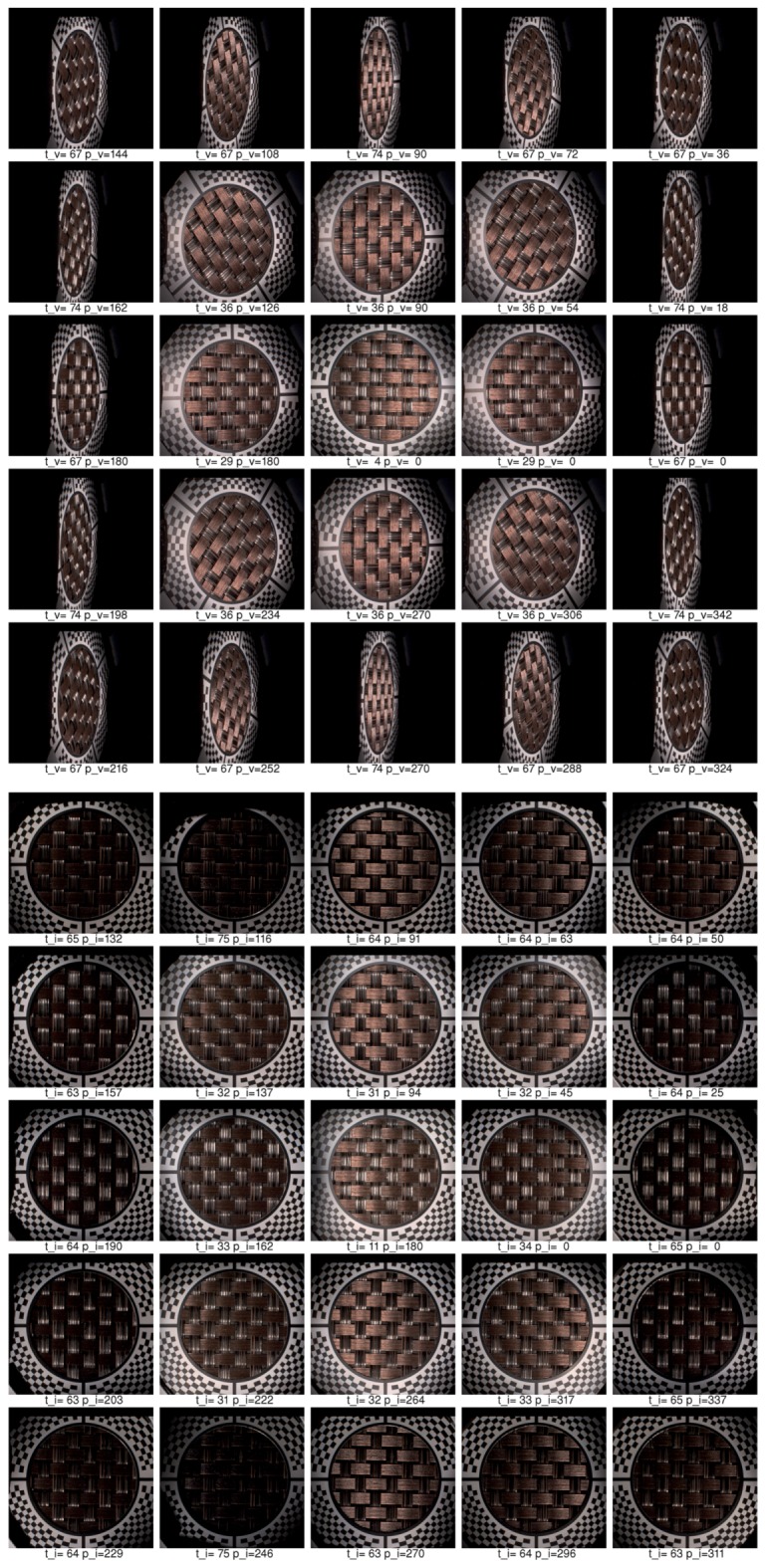
The example measurement data on example material sample 016-BasketWeave from The MAM 2014 sample set ([35]). (**top**) changing the viewing direction for illumination direction close to the normal for $\theta_{i}=12.5^{\circ }$; (**bottom**) changing the illumination direction, the camera at direction $\theta_{v}=3.5^{\circ }$, $\phi_{v}=1^{\circ }$.

**Figure 23:**
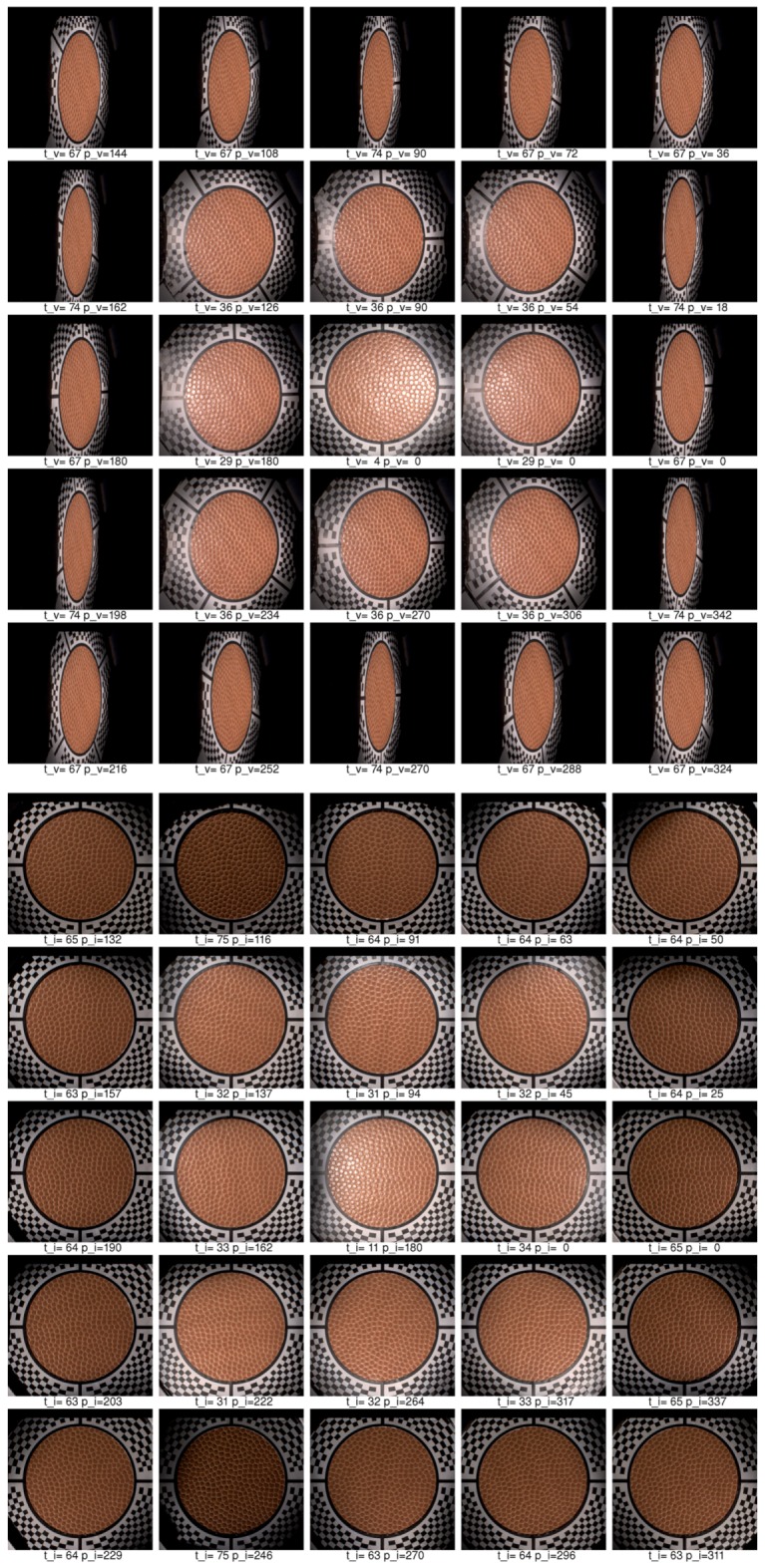
The example measurement data on example material sample 009-Basketball from The MAM 2014 sample set ([35]). (**top**) changing the viewing direction for illumination direction close to the normal for $\theta_{i}=12.5^{\circ }$; (**bottom**) changing the illumination direction, the camera at direction $\theta_{v}=3.5^{\circ }$, $\phi_{v}=1^{\circ }$.

**Figure 24:**
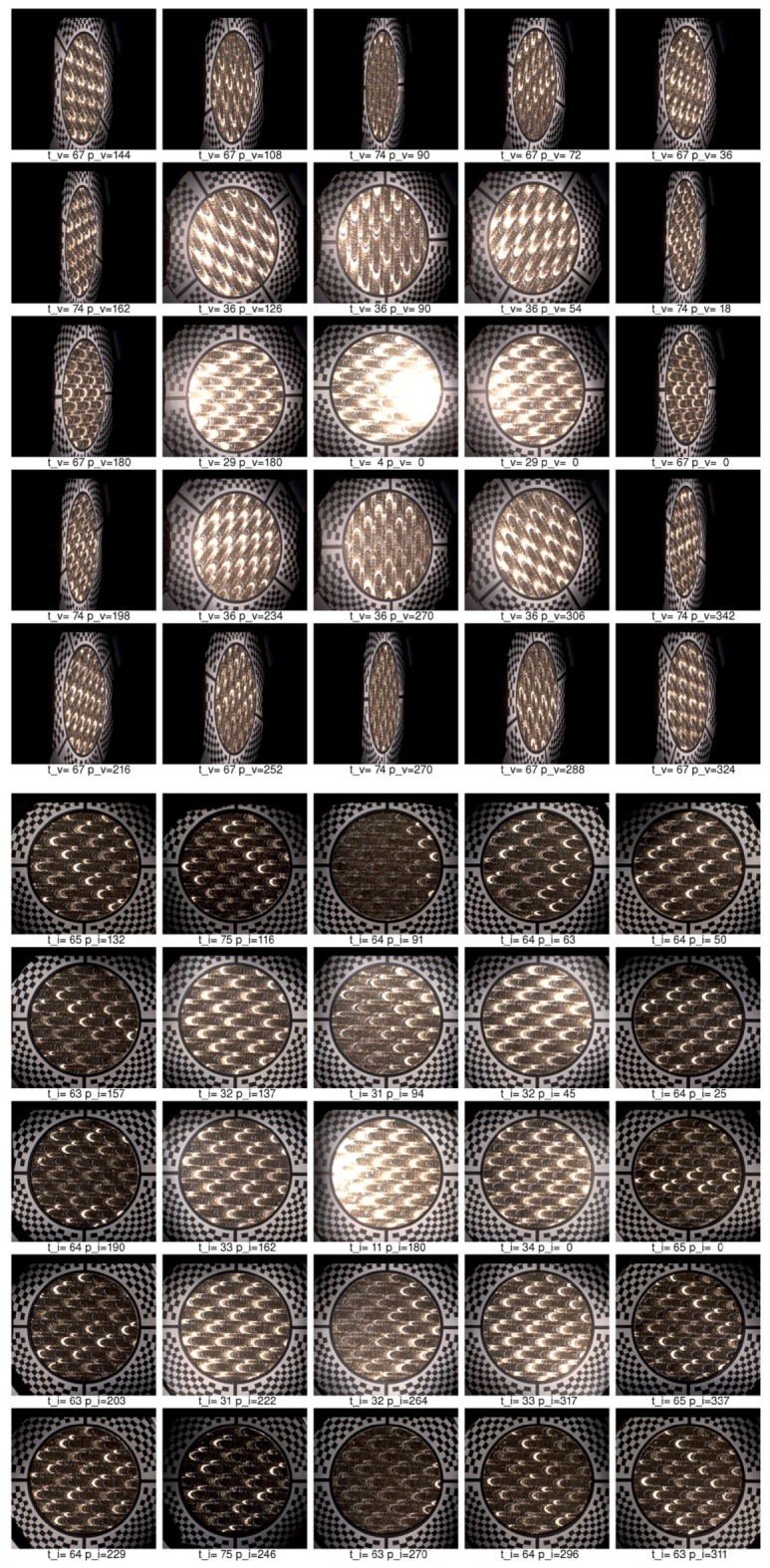
The example measurement data on example material sample 011-Silver-Gold from The MAM 2014 sample set ([35]). (**top**) changing the viewing direction for illumination direction close to the normal for $\theta_{i}=12.5^{\circ }$; (**bottom**) changing the illumination direction, the camera at direction $\theta_{v}=3.5^{\circ }$, $\phi_{v}=1^{\circ }$.

We ran over 60 measurements to find the important issues and get practical experience. Some material samples were measured twice to check the measurement reproducibility. Based on our experiences we came up with the description of the measurement setup procedure as described in Section 7.3. For debugging we first measured samples in laboratory conditions with the holder frame for the floor samples. After finishing the instrument debugging in the lab, we tested the device in situ for both frame holders. We measured different data at different locations and with different instrument orientations as shown in Figure 4. The example of data illustrating the measurement procedure for 3 material samples are shown in Figure 22, Figure 23 and Figure 24.

### 12.3. MAM 2014 Sample Set Measurements

**Figure 25:**
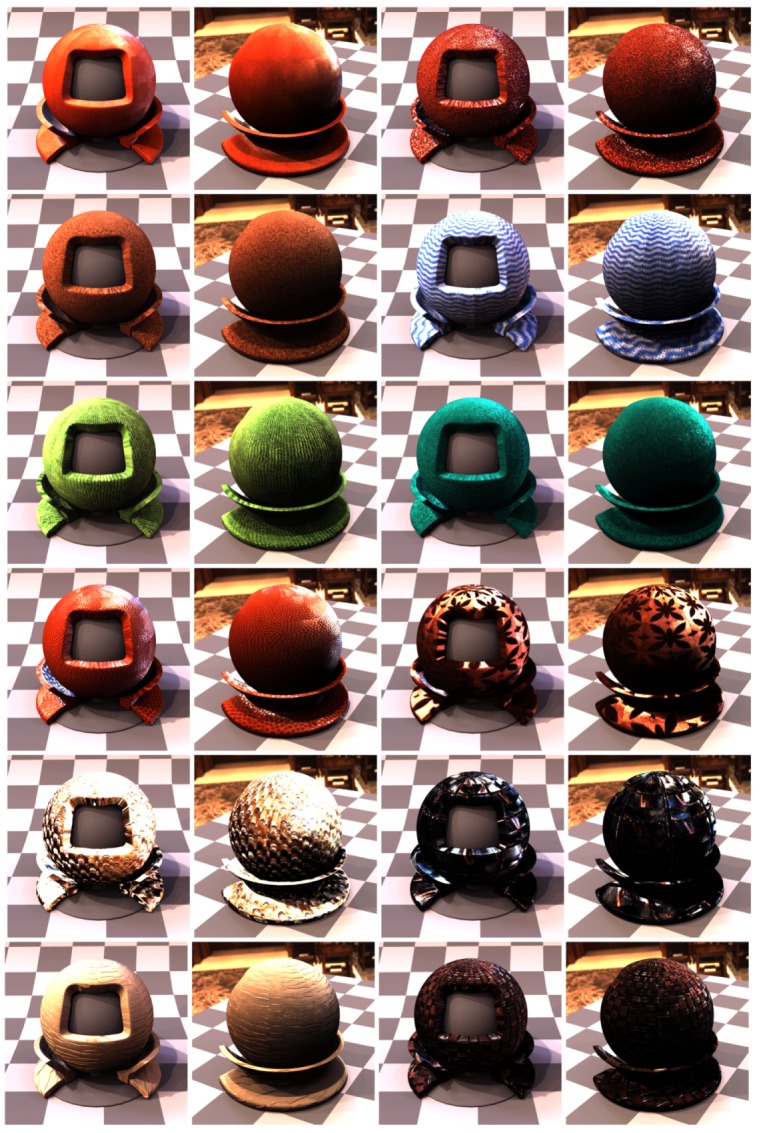
Twelve material samples from The MAM 2014 sample set, measured by lightdrum, put onto the 3D object and rendered by Mitsuba rendering software under two different views. Materials (top-left to bottom-right): 002-Sand-Fine, 003-Sand-Coarse, 005-Cork, 006-Towel, 007-GreenCloth, 008-GreenFelt, 009-Basketball, 010-FlockedPaper, 011-Silver-Gold, 014-Blue-Black-Gold, 015-Crinkle-Paper, 016-BasketWeave.

We also used for measurement 12 (out of 16) materials from The MAM 2014 sample set [35] that had a sufficient size, i.e., comparable or larger than the measurement hole diameter of 51 mm in the marker sticker. We next describe how we measured these samples. First, we glued the samples to the planar board. If the sample was smaller than the marker sticker (85×85 mm${}^{2}$), we put an additional construction around the sample to get a flat plane at the top surface of the sample. Then we glued the marker sticker onto the sample. We placed the sample under the gantry in the correct position and carried out the measurement. The resulting rendered images for MAM 2014 sample set are shown in Figure 25.

### 12.4. Data Processing

We transferred the data from the gantry to the computer and processed the measured data to get BTF as described in Section 11. Then we compressed the BTF by PCA as described in [36]. Also, we implemented and run the seamless spatial enlargement algorithm using published by Haindl and Hatka [37].

### 12.5. Rendering

**Figure 26:**
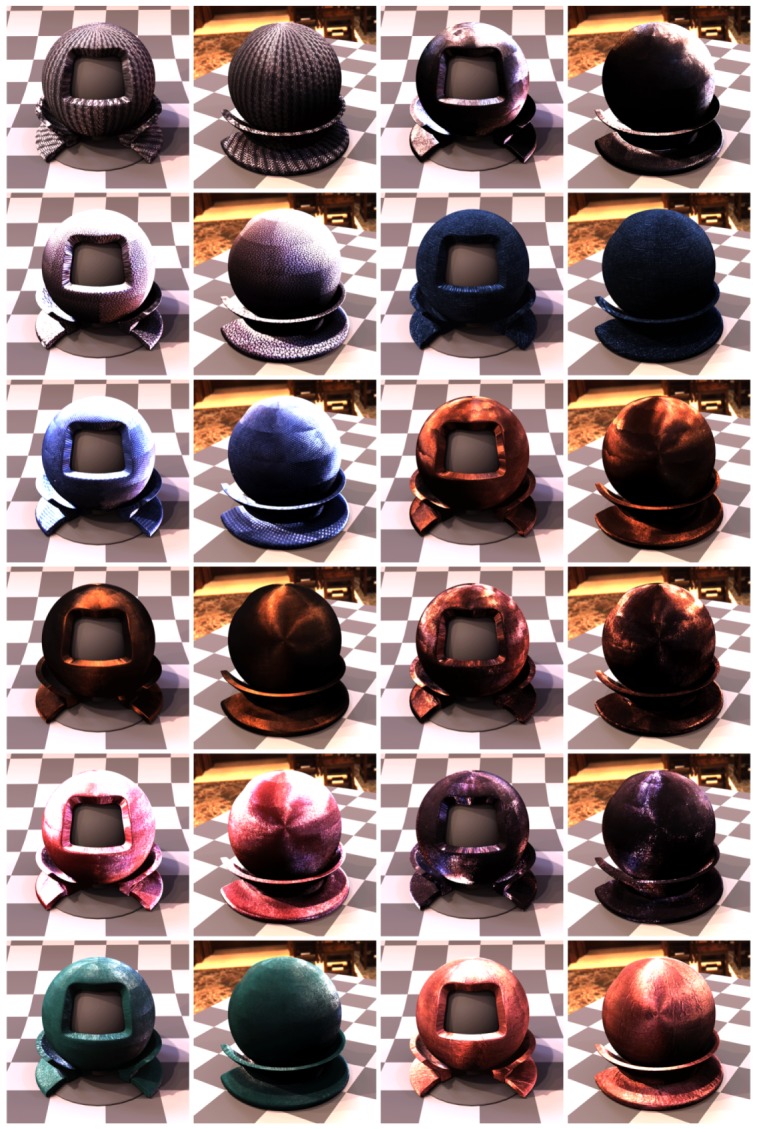
Twelve textile and upholstery material samples, measured by lightdrum, put onto the 3D object and rendered by Mitsuba rendering software under two different views. Materials (top-left to bottom-right): treves2, black leather, tres5, blue denim, fabric003, fabric111, fabric112, fabric135, fabric136, fabric137, fabric300, brocadi001. The materials fabric003, fabric111, fabric112, fabric135, fabric136, fabric137 are courtesy of Jiří Filip.

**Figure 27:**
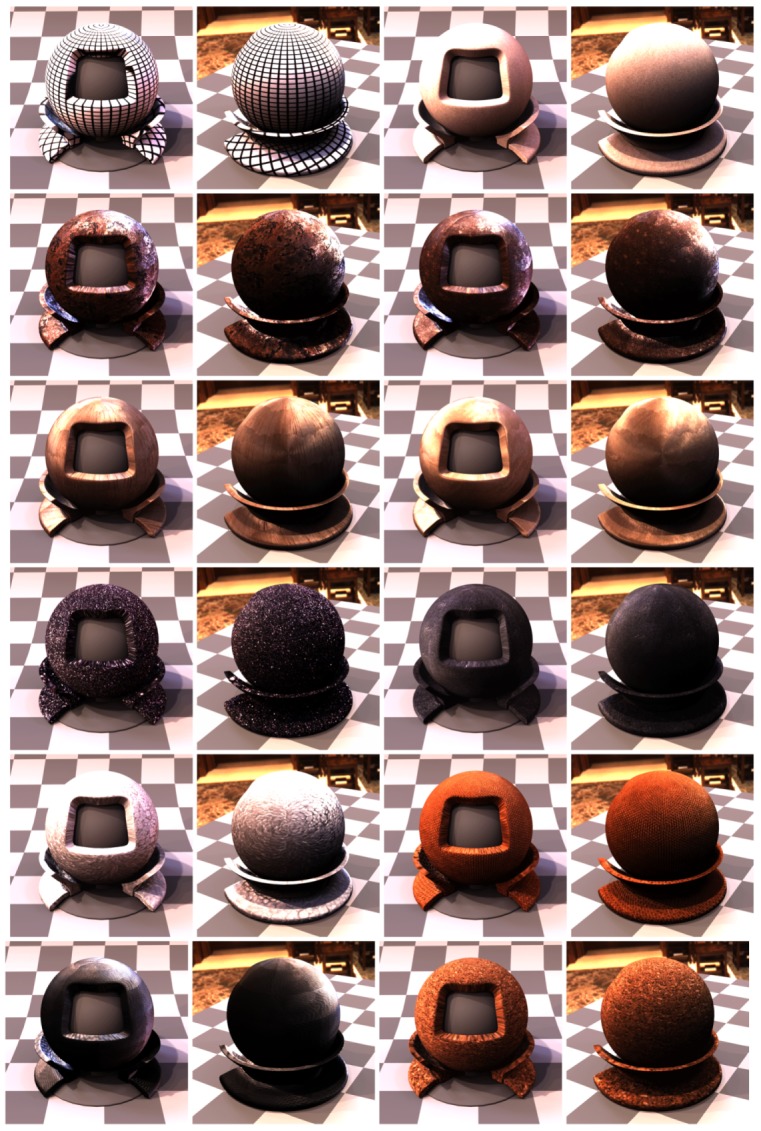
Twelve material samples from other categories, measured by lightdrum, put onto the 3D object and rendered by Mitsuba rendering software under two different views. Materials (top-left to bottom-right): office paper with printed grid 5×5 mm${}^{2}$ as used in demonstrations in Section 11, paper002, stone001, stone002, balsa wood, poplar wood, softening material 001, softening material 002, polystyrene coarse, hardboard, car upholstery, coarse cork.

**Figure 28:**
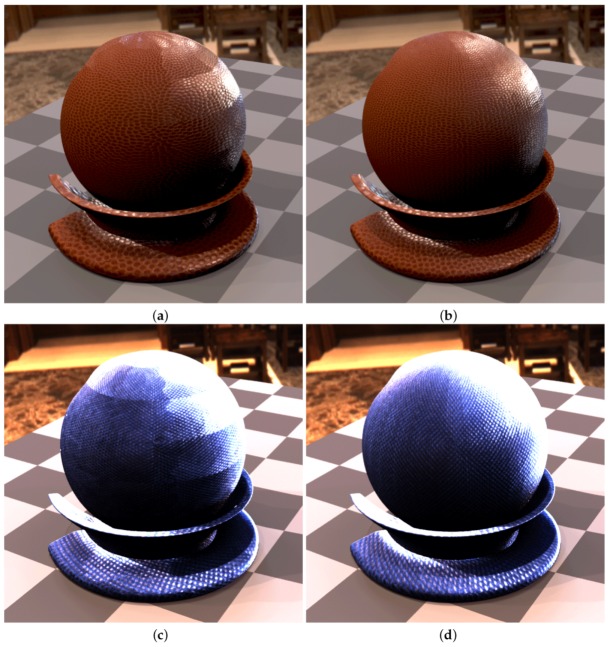
Overcoming the limitation of highly glossy materials by restricting the field of view—visible disturbing seams in directional domain on the tile. Material sample basketball from The MAM 2014 sample set: (**a**) the original tile size; (**b**) the decreased tile size usage. Measured material sample fabric003, physical sample courtesy of UTIA BTF Database: (**c**) the original tile size; (**d**) the decreased tile size.

We wrote a PCA decompression software plugin to the renderer Mitsuba [38] for environment map illumination. We have used for rendering the environment map *Grace-New* (http://gl.ict.usc.edu/Data/HighResProbes/probes/grace-new.hdr, courtesy of Paul Debevec). The rendered images for 36 measured material samples (selected out of 50 to present various materials, similar or diffuse material samples are not shown) are shown in Figure 25 (MAM 2014 sample set) and in Figure 26 (upholstery and textile) and in Figure 27 (other materials). To get the final images that are used in this paper (Figure 25, Figure 26, Figure 27 and Figure 28) we had to adjust the lightness of images, as the paper and display screens only exhibit a low dynamic range. To keep the background of images the same, we individually adjusted the intensity of BTF by a single multiplicative constant (darker materials are brighter, brighter materials are darker). This is necessary, if we want to depict the material features better, due to the dark and light adaption mechanism of the human eye, since it is impossible to transfer the high dynamic range data in the limited intensity range of computer output devices such as displays and printers. The rendered images are shown for applying the full sample size measured (the square 35.5×35.5 mm${}^{2}$ on the measured sample 200×200 pixels). We scaled the used BTF dataset 6 times in both U and V directions of the used UV texture mapping over the 3D object’s surface. For rendering we used far-field assumption for the measured BTF data, even though this is only an approximation, as neither the camera is orthographic nor is the illumination collimated. This may result in artifacts as we discuss further.

### 12.6. Comparison

Unfortunately, we do not have any stationary-type BTF gantry at our disposal in order to provide a reference measurement of the same data and to give a one-to-one direct comparison of the measured BTF data. All we had was three textile data from the project (UTIA BTF Database - BRDF Dense), namely material sample fabric112, fabric135, fabric136 that were measured as BRDF and one textile measured as BTF data (fabric03). All the four materials measured by UTIA gonioreflectometer are shown in Figure 26. The data from the stationary gonioreflectometer are available on the UTIA BTF Database website (http://btf.utia.cas.cz/). The samples from MAM 2014 sample set were distributed physically at the MAM 2014 conference in June 2014. To our knowledge we are the first to show the BTF measurement of this sample set, although only partially for 12 out of the 16 material samples.

Unfortunately, we cannot provide any radiometric measure of similarity between the data we have measured and the data measured by the stationary gonioreflectometer for any sample, as the details of radiometric and colourimetric calibrations of data measured on an UTIA stationary setup were not given. The methodology for comparison of two BTF datasets measured with different instruments has not yet been developed and published and it is more involved problem than for BRDF gantries (such as the method in [39]). It seems to be an interesting topic for further research.

### 12.7. Data and Videos

The demonstration of the lightdrum functionality during debugging and before the carbon cover has been mounted is shown in the accompanying video. The data and videos are available at the Supplementary Materials: http://dcgi.fel.cvut.cz/projects/lightdrum/.

## 13. Limitations

The presented BTF measurement device and methodology has some limitations that we would like to discuss here in detail.

### 13.1. Optical and Spatial Limitations

The first limitation is due to the physical laws of optics that we described in Section 5.1 and Appendix A that limits the image acquisition because of the depth of field. The spatial resolution in terms of lines per mm (or dots per inch) on the measured sample and the size of the measured sample are limited for the maximum zenith angle $\theta_{max}$ of the camera, in our case $\theta_{max}=75^{\circ }$. This maximum camera zenith angle is also frequently found in stationary BTF gantries as shown in the survey [4]. For measuring BRDF it could make sense to increase the $\theta_{max}$ angle to 80${}^{\circ }$. However, there is also another tradeoff given by the possibility of manipulating the gantry against the sample, since the gantry is terminated by the conic endpiece with the rectangular measurement aperture. The larger the angle $\theta_{max}$ the less space is available for manipulating the gantry over the sample. Another limitation is that we cannot approach a sample if it is closer than 300 mm to an obstacle or there is some concave region we cannot get into. The lightdrum must be able to freely rotate above the sample.

### 13.2. Camera Limitations

Our gantry uses only trichromatic cameras in RGB space with a Bayer mask which in theory could be changed to multi-spectral cameras if the footprint of these cameras is small enough. Another limitation comes from the length of time needed to take the measurement which is governed by the speed of HDR image acquisition for the used camera model, image processing (composing HDR image from 4 LDR images), and storing the HDR images onto the disks. The measurement time will be decreased by upcoming hardware technologies with newer and faster HDR camera models, communication interfaces, the raw processing power of mobile small sized embedded computers and the speed of external memories.

### 13.3. Angular Limitations of Illumination

Another limitation is the number of LED modules on the hemispherical module and between the cameras. From a practical point of view we limited the number of LED modules to 139 (134 + 5) which we found to be appropriate taking into consideration the accuracy of the existing stationary BTF gantries and the acceptable weight of the portable device. While we can position the cameras in arbitrary directions to a sample for the zenith angle up to 75${}^{\circ }$, the LED modules directions are always fixed in respect to the servo motor rotation. This is however not a severe limitation even compared to stationary dome based setups that use 151 LED modules (UBO Dome 1) and 198 LED modules (UBO Dome 2). For more details of UBO setups we refer to [4].

### 13.4. Highly Glossy BTF

As the portable instrument must have a small footprint, either the field of view or the size of the sample are limited. The camera field of view for the measured sample is $11.4^{\circ }$ given that the marker sticker hole diameter is 51 mm. The smaller the field of view the better. The same holds for illumination; in an ideal case the illumination can be made collimated. The large stationary setups can reach a smaller field of view with perspective lens imaging as the camera can be relatively far from the sample. In these setups this assumption is fulfilled only approximately; sometimes it is called far-field assumption. In smaller BTF measurement instruments, such as our lightdrum, for the measured samples containing moderately or highly glossy or specular surfaces, this field of view can be too high as glossy reflections could vary too much over the measured sample surface. This is visible, for example, in the rendered image in Figure 28a,c.

The first and simple method to resolve this is to use a smaller spatial image region from the measured data set around the measurement hole centre, as shown in Figure 28b,d, with a relatively small detrimental result of decreasing the visual variability and the richness of the rendered images. The second possible, more complex method to resolve this issue is software based. For each apparent BRDF for a texel at x,y we can specify all samples corresponding to the real geometry of imaging and illumination. The data for each apparent BRDF can then be resampled in four dimensions. This is best done in a double hemispherical parameterization without discontinuities. This resampling method was described in the paper by Ruiters et al. [40]. It is also in principle possible to combine both methods.

We also studied the possibility of using telecentric lenses (achieving in principle a field of view of $0^{\circ }$) while keeping the sample size the same. This is hardly possible, as it would require too much space for large telecentric lenses. It is interesting to compare the field of view of the lightdrum design with the existing stationary BTF dome based devices. The UBO Dome 1 device has a field of view of either $9.2^{\circ }$ and the UBO Dome 2 either $3.3^{\circ }$ or $12.6^{\circ }$, as documented in [4]. In this respect our portable instrument has a smaller sample size and a comparable field of view ($11.4^{\circ }$).

Our portable design provides a tradeoff; it allows both (a) to use a relatively large sample for up to moderately glossy surfaces; (b) to use only a smaller sample size for more glossy surfaces or the resampling method by Ruiters et al. [40]. The data for more glossy surfaces are aligned using the same marker sticker independently on the measured material sample. The decision to restrict the amount of the measured data to a smaller size or to resample the data is carried out after the measurement. The best such decision is taken after the visual checking of resulting rendered images. When we restrict ourselves to a smaller sample area, it requires us only to quickly recompute the spatial enlargement from smaller images, cropped to square around the centre from the full sized images. This recomputation of spatial enlargement using the algorithm [37] takes only a little time, in the order of tens of seconds.

### 13.5. Construction and Environmental Limitations

We optimised the design of the portable device to be lightweight. Without the servo motor with gear (1.8 kg) and the frame holder (2 kg) the critical weight of the rotational lightdrum excluding external cables is 11.2 kg. The weight of the service power box is 10 kg. Nevertheless, we believe that even with much more expensive technologies, as used e.g., in aerospace research, the lightdrum weight could be decreased by only about 15%. Another limitation comes from the power consumption. The lightdrum uses between 80 and 120 Watts in total during operation. The operating temperature range is between −10 ${}^{\circ }$C and +45 ${}^{\circ }$C. The operating humidity range was not tested, but we definitely cannot use the device in rather humid environments as the air humidity would condense on the camera lenses and possibly, the electronics cooled by the air ventilators would be affected or even damaged.

### 13.6. Other Limitations

There were many technical challenges that are, in fact, also a kind of limitation as the instrument has to be physically built. This mostly includes a sufficient mechanical rigidity while keeping the low weight of the device. It was also not easy to produce the hemispherical dome with the holes: we were not able to produce it from carbon as no company in our vicinity was willing to do CNC machining of a carbon hemisphere. Another limitation also comes from the camera drivers, as the chosen cameras must be compatible with the selected embedded microcomputers and they must allow for HDR acquisition at the same time.

## 14. Conclusions

We have proposed a new principle of a portable rotational light stage to measure surface reflectance in situ. The proposed solution, called a Lightdrum, achieves two arbitrary degrees of freedom for cameras moving over a stationary sample, while the illumination directions are fixed in relation to the cameras. This provides a suitable tradeoff between the complexity of mechanical construction and the speed of measurement, while allowing for a high degree of flexibility. The proposed Lightdrum is mainly for use in the applications of computer graphics and computer vision.

We designed, built, debugged, adjusted and tested a light weight Lightdrum prototype, including all the mechanical, optical, electronic and software components necessary for this project. This required careful interdisciplinary co-design. Our device consists of both custom made and off-the-shelf components. We tested the utility of the prototype starting with measurement in a lab and moving to real life on site scenarios. Some parameters of the concept and the performance of the system such as the measurement time will be improved on with better and faster computer and camera technologies.

In the future, we want to build a second lightdrum prototype to allow for the measurement of transparent and translucent materials on site. We also want to improve the performance of the system in terms of measurement time when new hardware becomes available. Further, we think that an interesting research direction is to find the optimal design of the proposed marker sticker with a circular hole for BTF/BRDF measurement on site, together with a corresponding fast algorithm for image registration. It could be also interesting to extend the technique by estimating the shape of the measured sample if it is only close to a plane.

## Figures and Tables

**Table 1:** Comparison of the two stationary dome based setups made by the University of Bonn and our proposed portable setup lightdrum. The table was adopted from [4], restricted only to BTF measurements and extended by several lines.

Parameter/Setup	Dome 1		Dome 2	Lightdrum
Configuration (Year)	2004	2008/2011	2012	2016
dimensions (L × W × H) [mm]	1900×1900×1900		3400×2500×2500	820×660×520 ${}^{1}$
distance to sample [mm]	650		1000	251
directions $\omega_{i}\times \omega_{o}$	151×151		198×264	139×120
resolution $\omega_{i}$	9.4${}^{\circ }\;\pm \;1^{\circ }$		9${}^{\circ }\;\pm \;1.2^{\circ }$	11.1${}^{\circ }\;\pm \;0.5^{\circ }$
resolution $\omega_{o}$	9.4${}^{\circ }\;\pm \;1^{\circ }$		7.6${}^{\circ }\;\pm \;2.6^{\circ }$	10.1${}^{\circ }\;\pm \;3.3^{\circ }$
maximum *θ*	75${}^{\circ }$		75${}^{\circ }$	75${}^{\circ }$
equivalent focal length ${}^{2}$ [mm]	116	104	190/95	80
focal length [mm]	16.22	22	100/50	12.5
spatial resolution [DPI]	235	450	380/190	300/150 ${}^{3}$
dynamic range ${}^{4}$ [dB]	28/33/33	25/44/44	32/60/*∞*	60.06/78.06/-
spectral bands	RGB		RGB	RGB
camera type	Canon P&S		Industrial CCD	Industrial CMOS
camera sensor size [mm]	5.312×3.984	7.44×5.38	16.67×16.05	5.20×3.88
camera sensor pixel resolution	2048×1536	4000×3000	2048×2048	2080×1552
#cameras	151		11	6
camera data	8 BPP JPEG		12 BPP raw	12 BPP raw
light source type	flash		LED	LED
#light sources	151		198	139
measurement size [mm]	105×105	105×105	75×75/140×140	⊘ 51
direction variation (field of view)	$9.2^{\circ }$		$3.3^{\circ }/12.6^{\circ }$	$11.4^{\circ }$
BTF raw/HDR images ${}^{5}$	91,204/22,801		156,816/52,272	66,720/16,680
saved HDR images resolution	2048×1536	4000×3000	2048×2048	1040×776
equivalent BTF HDR size [GB] ${}^{6}$	200	764	612	40 ${}^{3}$
BTF size (disk space) [GB]	22	281	918	40 ${}^{3}$
BTF time [h]	1.8		4.4–9.7 ${}^{7}$	0.28
HDR speed [Msamples/s] ${}^{8}$	10.56	40.27	6–13.2	12.58 ${}^{3}$
radiometric repeatability ${}^{9}$	-	7.4	0.1	<0.6${}^{10}$
geometric repeatability ${}^{11}$	-	0.81 px/0.006${}^{\circ }$	0.12 px/0.002${}^{\circ }$	${}^{12}\;\pm$1.20 px/0.011${}^{\circ }$
				and ±0.05 px/0.002${}^{\circ }$
sample flexibility	none		some; arbitrary $\phi_{o}$	arbitrary $\omega_{o}$, dependent $\omega_{i}$
radiometric calib. procedure	complex		easy	easy
geometric calib. procedure	automatic		automatic	automatic
durability (#measurements)	- ${}^{13}$	≈265/>347 ${}^{14}$	>3650 ${}^{15}$	>20,000 ${}^{16}$

${}^{1}$ Including the aluminium frame holder for floor measurements; ${}^{2}$ 35 mm equivalent focal length; ${}^{3}$ Stored for binning 2×2 or 4×4 sensor pixels, the latter fulfils the depth of field condition, larger images stored to the disk are used for image registration; ${}^{4}$ Single exposure/performed HDR measurements/ theoretical maximum; ${}^{5}$ Raw images taken by camera / stored HDR images, each consisting of 3 or 4 individual exposures; ${}^{6}$ Data from [4] were recomputed for the sake of consistency and to contain only surface reflectance data (no data for 3D reconstruction), Ward’s RGBE format for HDR data; ${}^{7}$ Time dependent on the material, the darker materials required longer exposures; ${}^{8}$ Raw speed computed from the texels of HDR images saved to the disk, the postprocessing of data to get BTF is not considered; ${}^{9}$ Given as variance in measured reflectivity for SphereOptics Zenith UltraWhite; ${}^{10}$ Given as worst variance in measured reflectivity for all camera/LED configurations for Fluorilon-99W${}^{\mathrm{TM}}$ produced by Avian Technologies, LED current was 100 mA; ${}^{11}$ Standard deviation in imaging condition in pixels/Standard deviation in angular configuration in degrees; ${}^{12}$ Worse geometric repeatability 0.034${}^{\circ }$ in azimuth direction for stepper motor with backlash ±0.05 mm, better one for geared servo motor, repeatability ±8 arcsec; ${}^{13}$ Not determined due to systematic defect of the CCD chips in the whole camera series; ${}^{14}$ Two camera CCDs became defective and were replaced after about 160,000 exposures. The other 149 are counting 210,000 exposures and are probably limited by the wear of the flashes; ${}^{15}$ Assuming one measurement per day. The camera manufacturer asserts continuous operation for at least 10 years. Test with the LEDs indicate a lifetime of at least 4000 measurements; ${}^{16}$ Minimum lifetime estimate based on the properties of motion mechanisms, LEDs, and cameras.

## Supplementary Materials

The data and videos are available at the following link: http://dcgi.fel.cvut.cz/projects/lightdrum/.

## Appendix A. DoF Derivation for BTF Instrument with Thin Lens Imaging

The following equations are derived from the geometry of thin lens imaging as depicted in Figure 5.

(A1) $$\begin{array}{c} \frac{-\Delta^{\prime }}{x^{\prime }-x_{A}^{\prime }}=\frac{D}{f^{\prime }+x_{A}^{\prime }} \end{array}$$

(A2) $$\begin{array}{c} \frac{-\Delta^{\prime }}{x_{B}^{\prime }-x^{\prime }}=\frac{D}{f^{\prime }+x_{B}^{\prime }} \end{array}$$

(A3) $$\begin{array}{c} DoF=x_{B}-x_{A} \end{array}$$

(A4) $$\begin{array}{c} -x+x^{\prime }+2f^{\prime }=l \end{array}$$

The Newtonian transfer equations give:

(A5) $$\begin{array}{c} x.x^{\prime }=-{\left(f^{\prime }\right)}^{2} \end{array}$$

(A6) $$\begin{array}{c} x_{B}x_{B}^{\prime }=-{\left(f^{\prime }\right)}^{2} \end{array}$$

(A7) $$\begin{array}{c} x_{A}x_{A}^{\prime }=-{\left(f^{\prime }\right)}^{2} \end{array}$$

The further two equations describe a lateral magnification:

(A8) $$\begin{array}{c} \frac{\Delta^{\prime }}{\Delta }=\frac{x^{\prime }+f^{\prime }}{x-f^{\prime }} \end{array}$$

(A9) $$\begin{array}{c} \frac{\Delta }{f^{\prime }}=\frac{-\Delta^{\prime }}{x^{\prime }} \end{array}$$

The diameter of the circle of confusion due to diffraction (for visible spectrum the mean wavelength λ=550 nm) is:

(A10) $$\begin{array}{c} \Delta^{\prime }=2.44\frac{\lambda }{D}\left(x^{\prime }+f^{\prime }\right) \end{array}$$

The requirement for the DoF is given by the size of measured sample and maximum zenith angle $\theta_{max}$ (for example $\theta_{max}=75^{\circ }$) of camera axis with respect to sample:

(A11) $$\begin{array}{c} DoF>y\sin\theta_{max} \end{array}$$

And finally, the spatial resolution in dots per inch (DPI) is given as:

(A12) $$\begin{array}{c} R=25.4/\Delta \end{array}$$

Below we describe the derivation of the optics starting with the thin lens equation. From Equations (A4) and (A5) we can derive the sensor distance from the image focal point:

(A13) $$\begin{array}{c} x^{\prime }=\frac{l-2f^{\prime }-\sqrt{{\left(2f^{\prime }-l\right)}^{2}-4{\left(f^{\prime }\right)}^{2}}}{2} \end{array}$$

From the diffraction limit Equation (A10) we then express the aperture diameter *D*:

(A14) $$\begin{array}{c} D=\frac{2.44\lambda (x^{\prime }+f^{\prime })}{\Delta^{\prime }} \end{array}$$

From Equations (A2) and (A6) we obtain the location of the nearest point in focus and the location of its image

(A15) $$\begin{array}{c} x_{B}^{\prime }=\frac{\Delta^{\prime }f^{\prime }+Dx^{\prime }}{D-\Delta^{\prime }}\\ x_{B}=\frac{-{\left(f^{\prime }\right)}^{2}}{x_{B}^{\prime }} \end{array}$$

Similarly, from Equations (A1) and (A7) we then get the furthest distance in focus from the object focal point:

(A16) $$\begin{array}{c} x_{A}^{\prime }=\frac{Dx^{\prime }-\Delta^{\prime }f^{\prime }}{\Delta^{\prime }+D}\\ x_{A}=\frac{{\left(f^{\prime }\right)}^{2}}{x_{A}^{\prime }} \end{array}$$

Then from Equations (A3), (A15) and (A16) we can compute the depth of field, from which we then compute the pixel size that is projected onto an object in focus (distance *l* from object plane to sensor plane), and finally we get the spatial resolution in DPI:

(A17) $$\begin{array}{c} DoF=x_{B}-x_{A},\;\Delta =\frac{\Delta^{\prime }f^{\prime }}{x^{\prime }},\;DPI=25.4/\Delta , \end{array}$$

on the condition that the ratio $\frac{DoF}{y\sin\theta_{max}}\approx 1$. If we do not utilise the whole sensor area then $\mathrm{DoF}>y\sin\theta_{max}$. This gives the restrictions on the possible and meaningful configurations given by a camera, lens, the size of measured sample *y*, and the angle $\theta_{max}$.

## Appendix B. Geometric Uncertainty and Accuracy of Illumination and Cameras

Below we describe the sources of error for angular position uncertainty of illumination sources and cameras.

### Appendix B.1. Evaluation of Angular Position Uncertainty of Illumination Source

We assume that the meridional and zonal angles of the illumination sources are affected by errors caused by the production and alignment uncertainties of individual mechanical and optical components. The illumination sources are composed of LED chip and condenser lens Ledil FA11905_TINA3-S mounted on an adjustable platform. This platform is attached to the liner with three M3 screws. This liner is glued to the pre-milled holes in the dome and this dome is adjusted in relation to the sample. We identified separate sources of random alignment uncertainties as listed in Table A1.

**Table A1:** The uncertainty specification of illumination source position.

Illumination Alignment Uncertainty	Meridional Direction	Zonal Direction
LED chip soldered to the platform	±0.1 mm	±0.1 mm
LED chip inclination	0–15${}^{\circ }$	0–15${}^{\circ }$
LED chip inclination compensated		
with condenser decentration	20–2.6 mm	20–2.6 mm
adjustment screws clearance	±0.2 mm	±0.2 mm
adjustable platform alignment range	$\pm 1.7^{\circ }$	$\pm 1.7^{\circ }$
LED chip shift by adjustment	±0.014 mm	±0.014 mm
liner clearance in the dome hole	±0.2 mm	±0.2 mm
dome misalignment during machining	±0.1cosθ mm	±0.1 mm
dome inclination towards sample	$\pm 0.07^{\circ }$	$\pm 0.07^{\circ }$
dome misalignment towards sample	±0.5cosθ mm	±0.5 mm

We used the decentration of a condenser lens to compensate for any soldered LED chip inclination towards the adjustable platform. The relation between the lens decentration *S* and the ray angle *δ* is given by Prentice’s rule: $\tan\delta =S/f^{\prime }$, where $f^{\prime }$ is the lens focal length. The adjustable platform with M3 screws spaced at 28 mm apart can adjust the illumination beam to the sample in the range of $\pm 1.7^{\circ }$. The platform tilt will cause the LED to shift by the value of *S* as given by the equation:

$$S=L-\sqrt{L^{2}+L\tan\delta },$$

where *δ* is the adjustable platform alignment range angle and *L* is the distance between adjusting screws.

All alignment uncertainties are realised at the distance $l^{\prime }=261$ mm from the sample centre and need to be recomputed to misalignment angles with the equation $\tan\delta_{i}=S/l^{\prime }$. In the zonal direction all errors are independent and the total angular uncertainty Φ is given by its linear combination proportional to the equation:

$$\Phi^{2}=\sum_{n}\delta_{i}^{2}$$

In the meridional direction some uncertainties are proportional to the illumination source pitch angle *θ*. These are the dome misalignment towards the sample and the dome misalignment during machining, proportional to the cosθ of the illumination source. The maximum total illumination source angular uncertainty Φ given by linear combination of individual uncertainties is $0.59^{\circ }$ mainly caused by the LED chip inclination compensation.

### Appendix B.2. Evaluation of Angular Position Uncertainty of Cameras

We evaluated the meridional and zonal position uncertainties of the cameras in a similar way to the case of the illumination source, taking into account all mounting and alignment uncertainties. The cameras are mounted on an adjustable platform which is fixed to the movable arm with three M3 screws. The arm is guided with a linear guide mount on one leg of the inner aluminium frame and driven with a stepper motor with a threaded screw. We identified separate sources of random alignment uncertainties as listed in Table A2.

**Table A2:** The uncertainty specification of camera position.

Camera Alignment Uncertainty	Meridional Direction	Zonal Direction
camera mount on adjustable platform	±0.2 mm	±0.05 mm
adjustment screws clearance	±0.2 mm	±0.2 mm
adjustable platform alignment range	$\pm 1.7^{\circ }$	$\pm 1.7^{\circ }$
camera shift by adjustment	±0.016 mm	±0.016 mm
fixtures mount on the arm	±0.04 mm	±0.04 mm
linear guide clearance	-	±0.015 mm
threaded screw axial end play	±0.3 mm	-
frame’s leg mounting position	±0.1 mm	±0.5 mm
inner aluminium frame misalignment during machining	±0.1cosθ mm	±0.1 mm
inner aluminium frame inclination towards sample	$\pm 0.07^{\circ }$	$\pm 0.07^{\circ }$
inner aluminium frame misalignment towards sample	±0.5cosθ mm	±0.5 mm

The adjustable platform with M3 screws spaced 25 mm apart can adjust the cameras to the sample in the range of $\pm 1.7^{\circ }$. This tilt will cause the cameras to shift by a value given by the equation:

$$S=L-\sqrt{L^{2}+L\tan\delta },$$

where *δ* is the adjustable platform alignment range angle and *L* is the distance between the adjusting screws.

All alignment uncertainties are realised at the distance l=251 mm from the sample and need to be recomputed to misalignment angles with the equation $\tan\delta_{i}=S_{i}/l$. In the zonal direction all errors are independent and the total angular uncertainty Φ is given by its linear combination proportional to the equation:

$$\Phi^{2}=\sum_{n}\delta_{i}^{2}.$$

In the meridional direction some uncertainties are proportional to the camera pitch angle *θ*. These are the inner aluminium frame misalignment towards the sample and the inner aluminium frame misalignment during machining which is proportional to the cosθ of the camera. The maximum camera total angular uncertainty Φ given by linear combination of individual uncertainties is $\pm 0.167^{\circ }$ in the meridional direction and $\pm 0.184^{\circ }$ in the zonal direction. The main effect to this uncertainty is caused mostly by the inner aluminium frame inclination towards sample uncertainty.

## Appendix C. Marker Sticker Code Listing in ANSI C++ Language

1 // *The source code written by Vlastimil Havran, September 2015*
  2 #include <cstdlib>
  3 #include <iostream>
  4 #include <fstream>
  5 #include <cmath>
  6 #include "Board.h"
  7
  8 using namespace LibBoard;
  9 using namespace std;
 10
 11 #define MIDCOLOR 0,255,0
 12
 13 // *Positive - printing black on white background*
 14 #define WHITE 255,255,255
 15 #define BLACK 0,0,0
 16 #define NOINVERT 1
 17 #define HOLE 1
 18
 19 inline float sqr(float a) { return a*a;}
 20
 21 // *From Libboard library https://github.com/c-koi/libboard by Sebastien Fourey*
 22 Board board;
 23
 24 void PutRectangle(float centerx, float centery, float width, float height)
 25 {
 26   board.fillRectangle(centerx-width/2.0, centery+height/2.0, width, height);
 27 }
 28
 29 int CropPoint(Point &p1, float mSize)
 30 {
 31   int cnt = 0;
 32   float dist = sqrt(p1.x*p1.x + p1.y*p1.y);
 33   float maxdist = sqrt(2.0)*mSize;
 34   bool crop = false;
 35   if (p1.x > mSize) {crop = true;}
 36   if (p1.x < -mSize) {crop = true;}
 37   if (p1.y > mSize) {crop = true;}
 38   if (p1.y < -mSize) {crop = true;}
 39
 40   if (crop) { // *Find the closest point to the center in the square*
 41     int cnt = 10000;
 42     for (int i = cnt; i ; i–) {
 43       float mult = (float)i/(float)cnt;
 44       Point pp = p1;
 45       pp.x *= mult;
 46       pp.y *= mult;
 47       bool crop2 = false;
 48       if (pp.x > mSize) {crop2 = true;}
 49       if (pp.x < -mSize) {crop2 = true;}
 50       if (pp.y > mSize) {crop2 = true;}
 51       if (pp.y < -mSize) {crop2 = true;}
 52       if (!crop2) {
 53         p1 = pp;
 54         return true; // *was cropped*
 55       }
 56     }
 57   }
 58   return false;
 59 }
 60
 61 int CropPointXY1(Point &pp, float mSizeX, float mSizeY)
 62 {
 63   int cnt = 0;
 64   if ((pp.x < mSizeX)&&(pp.y < mSizeY))
 65     cnt++;
 66   return cnt;
 67 }
 68
 69 int CropPointXY2(Point &pp, float mSizeX, float mSizeY)
 70 {
 71   int cnt = 0;
 72   if ((pp.x > mSizeX)&&(pp.y < mSizeY))
 73     cnt++; // *inside;*
 74   return cnt;
 75 }
 76
 77 bool CMP(float phi, float endPhi, int q)
 78 {
 79   if ((q % 2) == 0)
 80     return phi < endPhi;
 81   return phi > endPhi;
 82 }
 83
 84 template<class T> void Swap(T &x, T &y) { T temp = x; x = y; y = temp;}
 85
 86 int main(int argc, char *argv[])
 87 {
 88   board.clear( Color(WHITE) );
 89   float mSize = 85.f; // *mm - size of marker*
 90
 91   board.setLineWidth( 12.5 ).setPenColorRGBi( WHITE );
 92   board.setLineStyle( Shape::SolidStyle );
 93   board.setLineJoin( Shape::MiterJoin );
 94   board.setLineCap( Shape::RoundCap );
 95
 96   // *Draw a frame around the image - black*
 97   board.setLineWidth( 4.0 ).setPenColorRGBi( BLACK );
 98   board.drawRectangle(-0.5, 0.5, 1.0, 1.0);
 99   // *Draw a small frame right bottom*
100   board.setLineWidth( 1.5 ).setPenColorRGBi( BLACK );
101   board.drawRectangle(0.325, -0.43, 0.175, 0.07);
102   // *Draw a small frame left bottom*
103   board.setLineWidth( 1.5 ).setPenColorRGBi( BLACK );
104   board.drawRectangle(-0.5, -0.43, 0.092, 0.07);
105
106   // *chequerboard pattern in polar coordinates*
107   float deltaPhi = 2.9*M_PI/(64.0)/2.0; // *radians*
108   float deltaRad = 4.3f/mSize/2.0; // *mm*
109   const float phiStartOffset = 0.1f;
110   const float phiEndOffset = 0.08f;
111   // *Each quarter part of radial chequerboard patttern has its own data*
112   for (int q = 0; q < 4; q++) {
113     int odd2 = 0;
114     float begPhi, endPhi, stepPhi;
115     // *Set initial conditions*
116     if (q == 0) {
117       begPhi = phiStartOffset;
118       endPhi = M_PI/2.0 - phiEndOffset;
119       stepPhi = deltaPhi;
120     }
121     if (q == 1) {
122       begPhi = M_PI - phiStartOffset;
123       endPhi = M_PI/2.0f + phiEndOffset;
124       stepPhi = -deltaPhi;
125     }
126     if (q == 2) {
127       begPhi = M_PI + phiStartOffset;
128       endPhi = 3.0/2.0*M_PI - phiEndOffset;
129       stepPhi = deltaPhi;
130     }
131     if (q == 3) {
132       begPhi = 2.0*M_PI - phiStartOffset;
133       endPhi = 3.0/2.0*M_PI + phiEndOffset;
134       stepPhi = -deltaPhi*1.05;
135     }
136
137     // *Increasing phi*
138     for (float phi = begPhi; CMP(phi+stepPhi, endPhi, q); phi += stepPhi, odd2++) {
139      if (q == 0)
140         stepPhi *= 1.03f;
141       if (q == 1)
142         stepPhi = -deltaPhi*1.3 - deltaPhi*0.3*sin((phi-begPhi)*3.5 + M_PI*2.0f/4.0);
143       if (q == 2)
144         stepPhi = deltaPhi*1.3 + deltaPhi*0.3*sin((phi-begPhi)*5.0 + M_PI*1.25f/4.0);
145       if (q == 3)
146         stepPhi = -deltaPhi*1.3 + deltaPhi*0.3*cos((phi-begPhi)*5.5 + M_PI*1.25f/4.0);
147       int odd = 0; // *if to start with black or white region of chequerboard pattern*
148       if ((odd2%2) == 0) odd = 1;
149       // *Increasing radius by a fixed step*
150       for (float rad = 28.5/mSize; rad < 0.61; rad += deltaRad) {
151         int cnt = 0;
152         // *4 corners defining the radial region of the chequerboard pattern element*
153         Point p1(rad*cos(phi), rad*sin(phi));
154         Point p2((rad+deltaRad)*cos(phi), (rad+deltaRad)*sin(phi));
155         Point p3((rad+deltaRad)*cos(phi+stepPhi), (rad+deltaRad)*sin(phi+stepPhi));
156         Point p4(rad*cos(phi+stepPhi), rad*sin(phi+stepPhi));
157         if ((q == 1) || (q==3)) {
158           Swap(p2, p3);
159           Swap(p1, p4);
160         }
161         float maxf = 0.487;
162         cnt += CropPoint(p1, maxf);
163         cnt += CropPoint(p2, maxf);
164         cnt += CropPoint(p3, maxf);
165         cnt += CropPoint(p4, maxf);
166         float phi2 = atan2(p2.y, p2.x);
167         float phi3 = atan2(p3.y, p3.x);
168
169         float th = 0.004f;
170         float maxcheck = 0.48f;
171         if ( (q==0) && (p1.x > maxcheck) && (fabs(p1.x - p2.x) < th) &&
172              (fabs(p2.x - p3.x) < th) && (fabs(p4.x - p3.x) < th) ) continue;
173         if ( (q==0) && (p1.y > maxcheck) && (fabs(p1.y - p2.y) < th) &&
174              (fabs(p2.y - p3.y) < th) && (fabs(p4.y - p3.y) < th) ) continue;
175         if ( (q==1) && (p1.x < -maxcheck) && (fabs(p1.x - p2.x) < th) &&
176              (fabs(p2.x - p3.x) < th) && (fabs(p4.x - p3.x) < th) ) continue;
177         if ( (q==1) && (p1.y > maxcheck) && (fabs(p1.y - p2.y) < th) &&
178              (fabs(p2.y - p3.y) < th) && (fabs(p4.y - p3.y) < th) ) continue;
179         if ( (q==2) && (p1.x < -maxcheck) && (fabs(p1.x - p2.x) < th) &&
180              (fabs(p2.x - p3.x) < th) && (fabs(p4.x - p3.x) < th) ) continue;
181         if ( (q==2) && (p1.y < -maxcheck) && (fabs(p1.y - p2.y) < th) &&
182              (fabs(p2.y - p3.y) < th) && (fabs(p4.y - p3.y) < th) ) continue;
183         if ( (q==3) && (p1.x > maxcheck) && (fabs(p1.x - p2.x) < th) &&
184              (fabs(p2.x - p3.x) < th) && (fabs(p4.x - p3.x) < th) ) continue;
185         if ( (q==3) && (p1.y < -maxcheck) && (fabs(p1.y - p2.y) < th) &&
186              (fabs(p2.y - p3.y) < th) && (fabs(p4.y - p3.y) < th) ) continue;
187
188         if (cnt < 8) { // *We have not cropped the whole region, draw it*
189           // *Avoid right bottom corner*
190           const float bottomDistMarkY1 = -0.43;
191           const float bottomDistMarkX1 = 0.36;
192           int cnt2 = CropPointXY2(p1, bottomDistMarkX1, bottomDistMarkY1);
193           cnt2 += CropPointXY2(p2, bottomDistMarkX1, bottomDistMarkY1);
194           cnt2 += CropPointXY2(p3, bottomDistMarkX1, bottomDistMarkY1);
195           cnt2 += CropPointXY2(p4, bottomDistMarkX1, bottomDistMarkY1);
196           if (cnt2 > 0) {
197             continue;
198           }
199           // *Avoid left bottom corner*
200           const float bottomDistMarkY2 = -0.43;
201           const float bottomDistMarkX2 = -0.42;
202           cnt2 = 0;
203           cnt2 += CropPointXY1(p1, bottomDistMarkX2, bottomDistMarkY2);
204           cnt2 += CropPointXY1(p2, bottomDistMarkX2, bottomDistMarkY2);
205           cnt2 += CropPointXY1(p3, bottomDistMarkX2, bottomDistMarkY2);
206           cnt2 += CropPointXY1(p4, bottomDistMarkX2, bottomDistMarkY2);
207           if (cnt2 > 0) {
208             odd = !odd;
209             continue;
210          }
211           // *Draw a filed polygon of polar chequerboard region in black*
212           vector<Point> polyline;
213           // *First vertex p1*
214           polyline.push_back(p1);
215           cnt2 = 200; // *how finely decompose arc to line segments*
216           // *From p2 to p3*
217           float rad1 = sqrt(p2.x*p2.x + p2.y*p2.y);
218           float rad2 = sqrt(p3.x*p3.x + p3.y*p3.y);
219           float phi1 = atan2(p2.y, p2.x);
220           float phi2 = atan2(p3.y, p3.x);
221           int i = 0;
222           if (phi2 > phi1) {
223             for (float p = phi1; p < phi2; p += (phi2-phi1)/(float)cnt2, i++) {
224               float alpha = (float)i/(float)cnt2;
225               float r = rad1*(1.0 - alpha) + rad2*alpha;
226               Point pp(r*cos(p), r*sin(p));
227               polyline.push_back(pp);
228             }
229           } else {
230             for (float p = phi1; p > phi2; p -= (phi1-phi2)/(float)cnt2, i++) {
231               float alpha = (float)i/(float)cnt2;
232               float r = rad1*(1.0 - alpha) + rad2*alpha;
233               Point pp(r*cos(p), r*sin(p));
234               polyline.push_back(pp);
235             } // *for i*
236           }
237           polyline.push_back(p3);
238           // *From p4 to p1*
239           rad1 = sqrt(p4.x*p4.x + p4.y*p4.y);
240           rad2 = sqrt(p1.x*p1.x + p1.y*p1.y);
241           phi1 = atan2(p4.y, p4.x);
242           phi2 = atan2(p1.y, p1.x);
243           i = 0;
244           if (phi2 > phi1) {
245             for (float p = phi1; p < phi2; p += (phi2-phi1)/(float)cnt2, i++) {
246               float alpha = (float)i/(float)cnt2;
247               float r = rad1*(1.0 - alpha) + rad2*alpha;
248               Point pp(r*cos(p), r*sin(p));
249               polyline.push_back(pp);
250             }
251           } else {
252             for (float p = phi1; p > phi2; p -= (phi1-phi2)/(float)cnt2, i++) {
253               float alpha = (float)i/(float)cnt2;
254               float r = rad1*(1.0 - alpha) + rad2*alpha;
255               Point pp(r*cos(p), r*sin(p));
256               polyline.push_back(pp);
257             } // *for i*
258           }
259           if (odd) // *draw only black regions of polar chequerboard pattern*
260             board.fillPolyline(polyline, -1);
261         }
262         odd = !odd; // *change the order of black and white the next time*
263       } // *rad*
264     } // *phi*
265   } // *for q*
266
267   // *Add four rectangles to represent cross hair*
268   float bw = 0.02;
269   float bh = 0.18;
270   const float offsetMark = 0.40;
271   float kw = 1.0;
272   float kh = 1.0;
273   // *top middle*
274   PutRectangle(0.0, offsetMark, bw*kw, bh*kh);
275   // *bottom middle*
276   PutRectangle(0.0, -offsetMark, bw*kw, bh*kh);
277   // *right middle*
278   PutRectangle(offsetMark, 0, bh*kh, bw*kw);
279   // *left middle*
280   PutRectangle(-offsetMark, 0, bh*kh, bw*kw);
281
282   const float RADIUS = 23.5; // *mm*
283   const float RADIUS2 = 20.0; // *mm*
284   const float RADIUS3 = 51.0/2.0f; // *mm*
285   // *Draw the important circle by black color*
286   if (!HOLE) {
287     if (NOINVERT) {
288       // *black*
289       board.setLineWidth( RADIUS2 ).setPenColorRGBi( BLACK );
290       board.drawCircle(0, 0, 1.00*RADIUS/mSize, -1);
291     }
292     else {
293       float RADIUS3 = RADIUS + RADIUS2/6.0 + 0.215f;
294       board.setLineWidth(0.f).setPenColorRGBi( BLACK );
295       board.fillCircle(0, 0, RADIUS3/mSize,-1);
296     }
297   } else {
298     // *Make a hole in special color*
299     board.setLineWidth( RADIUS2 ).setPenColorRGBi( BLACK );
300     board.drawCircle(0, 0, 1.00*RADIUS/mSize,-1);
301     board.setLineWidth(0.f).setPenColorRGBi( MIDCOLOR );
302     board.fillCircle(0, 0, RADIUS3/mSize,-1);
303   }
304
305   // *Draw a cross in the middle of the sticker - black*
306   if (NOINVERT) {
307     // *This makes no sense for a sticker production company*
308     board.setLineWidth( 0.5 ).setPenColorRGBi( BLACK );
309     float deltaCross = 0.04f;
310     board.drawLine( -deltaCross, 0, deltaCross, 0);
311     board.drawLine( 0, -deltaCross, 0, deltaCross);
312   }
313
314   board.setPenColor( Color(BLACK) ).setFont( Fonts::HelveticaBold, 5.5 )
315     .drawText( -0.488, 0.440, "FEL FSI" );
316   board.setPenColor( Color(BLACK) ).setFont( Fonts::HelveticaBold, 5.5 )
317     .drawText( -0.488, 0.466, "CVUT" );
318   board.setPenColor( Color(BLACK) ).setFont( Fonts::HelveticaBold, 5.5 )
319     .drawText( 0.380, 0.466, "HAVRAN" );
320
321   // *85x85mm - final size of EPS/FIG/SVG*
322   board.saveEPS("markersticker.eps", mSize, mSize, 0);
323   return 0;
324 }

stickerlisting.cpp

## Appendix D. Gantry Assembly

We show the assembly of the lightdrum prototype in the series of 30 steps, each shown for three different camera viewpoints, in Figure A1, Figure A2, Figure A3, Figure A4 and Figure A5.
